# Complementary Activity of ETV5, RBPJ, and TCF3 Drives Formative Transition from Naive Pluripotency

**DOI:** 10.1016/j.stem.2019.03.017

**Published:** 2019-05-02

**Authors:** Tüzer Kalkan, Susanne Bornelöv, Carla Mulas, Evangelia Diamanti, Tim Lohoff, Meryem Ralser, Sjors Middelkamp, Patrick Lombard, Jennifer Nichols, Austin Smith

**Affiliations:** 1 Wellcome – MRC Cambridge Stem Cell Institute, University of Cambridge, Cambridge CB2 1QR, UK; 2 Department of Physiology, Development and Neuroscience, University of Cambridge, Downing Street, Cambridge CB2 3DY, UK; 3 Department of Molecular Biology, Faculty of Science, Radboud University, 6525GA Nijmegen, the Netherlands; 4 Department of Biochemistry, University of Cambridge, Tennis Court Road, Cambridge CB2 1GA, UK

**Keywords:** pluripotency, embryonic stem cell, epiblast, self-renewal, differentiation, commitment, gene regulatory network, ETS factors, RBPJ

## Abstract

The gene regulatory network (GRN) of naive mouse embryonic stem cells (ESCs) must be reconfigured to enable lineage commitment. TCF3 sanctions rewiring by suppressing components of the ESC transcription factor circuitry. However, TCF3 depletion only delays and does not prevent transition to formative pluripotency. Here, we delineate additional contributions of the ETS-family transcription factor ETV5 and the repressor RBPJ. In response to ERK signaling, ETV5 switches activity from supporting self-renewal and undergoes genome relocation linked to commissioning of enhancers activated in formative epiblast. Independent upregulation of RBPJ prevents re-expression of potent naive factors, TBX3 and NANOG, to secure exit from the naive state. Triple deletion of *Etv5*, *Rbpj*, and *Tcf3* disables ESCs, such that they remain largely undifferentiated and locked in self-renewal, even in the presence of differentiation stimuli. Thus, genetic elimination of three complementary drivers of network transition stalls developmental progression, emulating environmental insulation by small-molecule inhibitors.

## Highlights

• Downstream of pErk, ETV5 initiates GRN handover during ESC transition
• RBPJ enforces naive pluripotency exit by preventing re-expression of NANOG or TBX3
• ETV5 relocates to commission formative pluripotency enhancers
• ESCs lacking TCF3, ETV5, and RBPJ are trapped in a naive but nullipotent condition

## Graphical Abstract

**Figure:**
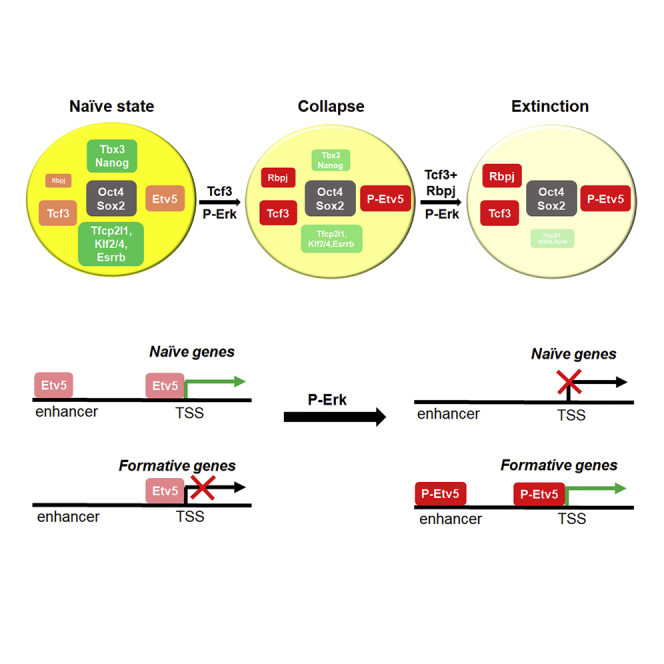

## Teaser

Smith, Kalkan, and colleagues report that the gene regulatory network in naive mouse embryonic stem cells is reconfigured to enable lineage commitment by combined action of two repressors, TCF3 and RBPJ, that dissolve and extinguish, respectively, the naive network and an activator, ETV5, that switches activity from supporting self-renewal and undergoes genome relocation linked to commissioning of enhancers in formative epiblast.

## Introduction

Mouse embryonic stem cells (ESCs) are *in vitro* cell lines that retain a high degree of molecular and functional correspondence with the naive pluripotent epiblast of the pre-implantation embryo (Boroviak et al., 2014, Bradley et al., 1984, Evans and Kaufman, 1981, Martin, 1981). Accordingly, they provide a rich resource for studying mechanisms underlying developmental decisions and transitions. In particular, the ESC pathway to differentiation *in vitro* provides an opportunity to dissect the progression of pluripotency from naive founder cells through to specification of germline and somatic lineage progenitors.

Culture in the presence of two small molecule inhibitors (2i) that suppress the MEK/Erk pathway and glycogen synthase kinase-3 (GSK3) sustains stable expression of transcription factor components of the naive pluripotency gene regulatory network (GRN) (Dunn et al., 2014, Wray et al., 2010, Ying et al., 2008). ESCs in these serum-free conditions are proposed to reside in a regulatory ground state (Ying et al., 2008). Upon release from 2i, ESCs transition into a distinct second stage of pluripotency that we have termed “formative” (Kalkan and Smith, 2014, Smith, 2017). Formative pluripotent cells have lost GRN components diagnostic of naive pluripotency and gained transcription factors characteristic of the peri-implantation epiblast, such as POU3f1, OTX2, and LEF1. Functional ESC identity is extinguished concomitant with change in transcription factor complement (Kalkan et al., 2017). In parallel, epigenetic processes, such as DNA methylation, are upregulated, and competence is gained for lineage induction (Hayashi et al., 2011, Mulas et al., 2017) and onward progression to primed pluripotency. The naïve-to-formative conversion in a simple and well-defined culture environment simulates events in the peri-implantation mouse embryo (Kalkan et al., 2017) and provides a sensitized platform for identifying factors and mechanisms that mediate change in cell identity (Buecker et al., 2014, Kalkan and Smith, 2014).

Genetic screens have identified several genes that promote ESC transition (Betschinger et al., 2013, Leeb et al., 2014, Li et al., 2018, Villegas et al., 2019, Yang et al., 2012). TCF3 (gene name *Tcf7l1*) was the first factor identified (Guo et al., 2011b) and is recurrently recovered. TCF3 represses key naive transcription factors ESRRB, TFCP2L1, NANOG, and KLF2 (Martello et al., 2012, Pereira et al., 2006), an effect blocked by GSK3 inhibition in 2i culture (Wray et al., 2011). Other pathways and factors identified in the screens have also been shown to reduce expression or function of components of the naive GRN, although to a lesser extent than TCF3. Strikingly, the majority of these components are present in naive ESCs but are ineffective in 2i (Kalkan and Smith, 2014). The pre-existence of multi-layered dissolution machinery means that mouse ESCs are poised for rapid disabling of the naive network. Multiple effectors also explain why single-factor mutants only delay and do not prevent transition.

However, although elimination of naive factors is necessary for departure from the ESC state, it may not be sufficient for installation of an alternative GRN, which requires new transcription factor expression and enhancer reconfiguration (Buecker et al., 2014, Factor et al., 2014, Yang et al., 2014). ERK pathway inhibition is the second component of 2i. ERK1/2 signaling likely contributes directly to naive GRN destabilization (Jin et al., 2016, Kim et al., 2014, Yeo et al., 2014), but its role in ESC transition to multilineage competence (Kunath et al., 2007, Stavridis et al., 2007) is also anticipated to include transcriptional activation (Tee et al., 2014, Williams et al., 2015).

Here, we sought to characterize drivers of the naive to formative transition that might act in parallel with TCF3 and examine whether genetic deletions might replace 2i and maintain naive ESC self-renewal.

## Results

### Identification of ETV5 as a Candidate Driver of Progression from Naive to Formative Pluripotency

To identify factors that may mediate the effect of ERK pathway inhibition in driving pluripotency network transition, we inspected results from loss-of-function screens. Among transcription factors, we noted that *Etv5* is the most recurrent hit after *Tcf3* in a random mutagenesis screen (Leeb et al., 2014) and is a high-confidence candidate from a genome-wide small interfering RNA (siRNA) screen (Yang et al., 2012). ETV5 is a member of the PEA3 sub-family of ETS transcription factors, along with Etv1 and Etv4 (Hollenhorst et al., 2011b, Oh et al., 2012). ETV5 and other ETS factors are typically activated by fibroblast growth factor (FGF)-ERK signaling through transcriptional upregulation and/or protein phosphorylation (Janknecht et al., 1996, Oh et al., 2012, Selvaraj et al., 2015). ETV5 is considered to be functionally redundant with ETV4, and the two factors are co-expressed in multiple tissues in response to FGF (Liu et al., 2003, Mao et al., 2009, Zhang et al., 2009) or glial cell line-derived neurotrophic factor (Lu et al., 2009).

**Figure 1:**
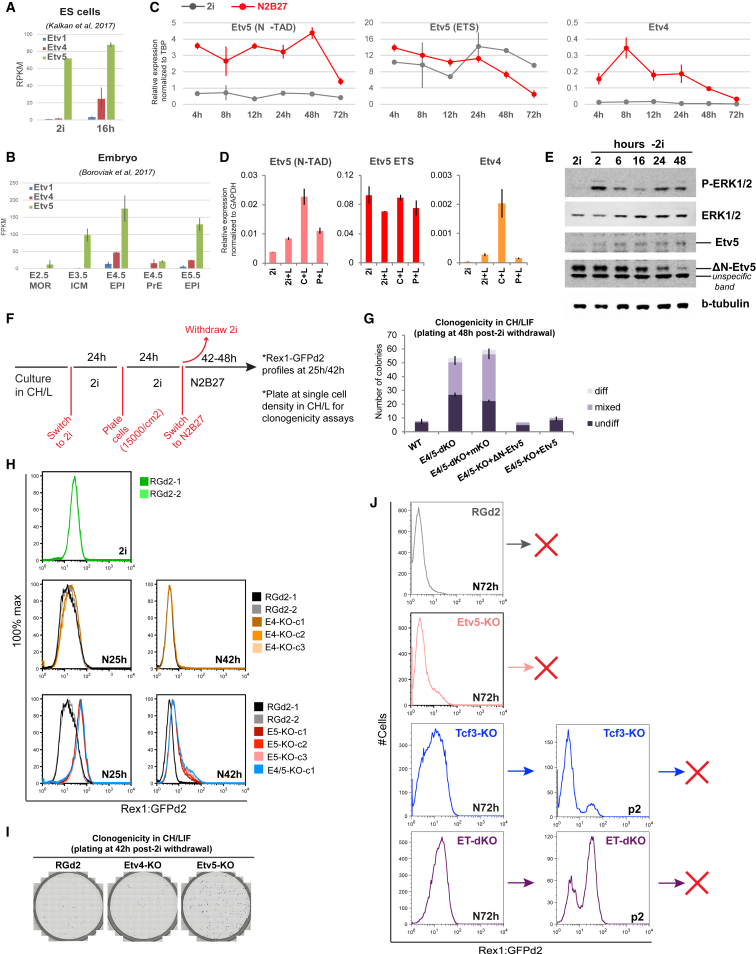
ETV4/5 Expression and Function (A and B) RNA-seq expression values (FPKM or RPKM) for naive and transitioning ESCs (A) and early embryo lineages (B). Error bars represent SD from (A) 2 and (B) 3 independent biological replicates. (C and D) qRT-PCR expression in 2i and after 2i withdrawal (C) and in 2i/L components for 3 passages (D). Data are means ± SD from (C) 2 wells of cells differentiated in parallel and (D) 3 independent biological replicates. CH, CHIR99021; L, LIF; P, PD0325901. (E) Western blot with anti-FLAG antibody on Etv5-3×FLAG knockin ESCs. (F) Schematic for (G)–(I). (G) Quantitation of colony assays on WT (wild type), *Etv4/5*-dKO ESCs, and *Etv4/5*-dKO ESCs expressing monomeric Kusabira Orange (mKO), ΔN-*Etv5*, or canonical *Etv5* transgenes. Error bars show SD from 2 technical replicates. (H) Rex1-GFP profiles of RGd2 and independently generated clonal lines (c) of Etv4-KO; Etv5-KO and Etv4/5-dKO at 25 and 42 h post-2i withdrawal. RGd2-2 is a clonal line derived from parental RGd2-1. (I) Colony assay. (J) GFP profiles for parental RGd2 and mutant ESC lines at 72 h post-CH/LIF withdrawal (N72h) and end of passage 2 (p2). Red cross indicates failure of replating after passage. ET, Etv5/Tcf3. See also Figures S1 and S2.

*Etv5* transcripts are readily detected in ground state ESCs (Figure 1A). In contrast, *Etv4* mRNA is not evident in 2i but is rapidly upregulated in transitioning cells. Transcripts for both factors are detected in mouse naive (embryonic day 4.5 [E4.5]) and formative (E5.5) epiblast, with ETV5 being more abundant (Figure 1B). *Etv1* expression is negligible in ESCs and the early embryo. We examined in closer detail the behavior of *Etv5* and *Etv4* in ESCs upon transfer from 2i to N2B27. Ribosome profiling indicated potential for a truncated ETV5 protein isoform (ΔN-Etv5) lacking the first 202 amino acids (Ingolia et al., 2011) that include an N-terminal transactivation domain (N-TAD) (Defossez et al., 1997, Laget et al., 1996). Accordingly, we designed alternative qRT-PCR primer pairs. We detected the ETS domain, but not the N-TAD encoding sequence, in undifferentiated ESCs, indicating expression of ΔN-ETV5 only. In contrast, N-TAD-containing transcripts appear within 4 h after 2i withdrawal and persist for 48 h (Figure 1C). Total *Etv5* transcripts increase initially but decline from 24 h, implying downregulation of ΔN-ETV5. *Etv4* expression also rises rapidly on removal of 2i and then reduces. We investigated steady-state *Etv4/5* expression in ESCs maintained with single inhibitors and LIF (Figure 1D). Both canonical *Etv5* and *Etv4* mRNA are upregulated in conditions when ERK signaling is active.

Inspection of RNA sequencing (RNA-seq) and H3K4me3 chromatin immunoprecipitation (ChIP)-seq data substantiated activity of an internal *Etv5* promoter in undifferentiated ESCs, whereas transcripts originating from the upstream start site are evident after 2i withdrawal (Figure S1A). Immunoblotting identified a smaller ΔN-Etv5 protein (Figures 1E and S1B) that decreased from 24 h of transition. Conversely, the canonical isoform was absent from undifferentiated cells and upregulated over the transition time course. Single-inhibitor withdrawal showed that canonical Etv5 is expressed in response to MEK and ERK activation. siRNA-mediated knockdown confirmed the specificity of immunoblotting (Figure S1B).

### Etv5 Fulfills Distinct Roles in ESC Self-Renewal and Transition

Due to the proposed redundancy between *Etv5* and *Etv4*, we first sourced ESCs genetically deficient for both genes (E4/5-double knockout [dKO]; Lu et al., 2009). These cells were derived in serum and LIF and reported to show reduced proliferation (Akagi et al., 2015). This phenotype is exacerbated in 2i/LIF (Figure S1C). However, we found that the mutant cells could be expanded robustly by omitting the MEK inhibitor and culturing in CH/LIF (Figure S1C). We therefore maintained E4/5-dKO cells in CH/LIF, but for consistency with previous studies (Kalkan et al., 2017, Mulas et al., 2017), cells were exchanged into 2i prior to assay. A short period of 2i culture has no apparent effect on growth rate or viability (Figure S1C). The assay entails withdrawal of 2i for 48 h before replating at clonal density in CH/LIF (Figure 1F). Self-renewal capacity is almost entirely extinguished in parental ESC by 48 h. In contrast, E4/5-dKO cells still generate numerous undifferentiated colonies (Figure 1G). This phenotype is eliminated upon expression of cDNA encoding either isoform of ETV5 (Figure 1G), both of which also rescue the growth defect during self-renewal (Figure S1C).

To discriminate functions of Etv4 and Etv5, we employed CRISPR/Cas9 to create single and double knockouts. We used RGd2 ESCs that carry the *Rex1::GFPd2* knockin reporter of naive status (Kalkan et al., 2017). Etv5-KO ESCs also show reduced expansion in 2i or 2i/LIF but proliferate normally in CH/LIF (Figure S1D). These results indicate that Etv5 plays a specific role in consolidating naive ESC propagation when ERK signaling is blocked and Etv4 is not expressed. We expanded cells in CH/LIF and transferred into 2i before assay, as above. *Etv4* mutants show no significant delay in GFP downregulation (Figure 1H) or extinction of clonogenicity (Figure 1I). In contrast, deletion of *Etv5* results in impaired exit from naive pluripotency, measured by perdurance of GFP and persistence of clonogenic cells. Normal GFP downregulation was restored by expressing either Etv5 isoform (Figure S1E). In *Etv5* mutants, Etv4 is activated later but to an enhanced level (Figure S1F). However, the Etv5-KO phenotype is not enhanced in *E4/5*-dKO ESCs (Figure 1H), confirming that Etv4 has little relevance for kinetics of transition from 2i.

These results establish that Etv5 supports ESC self-renewal when ERK signaling is inhibited and facilitates exit from naive pluripotency when ERK is active. Although canonical Etv5 is specifically upregulated prior to exit, either isoform can be sufficient for both functions.

### Co-deletion of *Etv5 and Tcf3* Retards, but Does Not Prohibit, Exit from Naive Pluripotency

*Tcf3* is upregulated in Etv5-KO ESCs, indicating that the phenotypes are independent (Figures S2A and S2B). As *Tcf3* is downstream of GSK3 and ETV5 is regulated by ERK1/2, we tested whether combined deletion of both genes might mimic the effect of 2i and be sufficient to sustain ESC self-renewal. We generated *Etv5/Tcf3* single and double mutants in RGd2 ESCs and compared GFP profiles after transfer into N2B27 (Figure 1J). In ET-dKO cells, perdurance of GFP was more pronounced. However, at the end of passage 2, ET-dKO cells showed a substantial fraction of GFP low or negative cells, and undifferentiated ESCs were not sustained after replating (Figure 1J). Thus, TCF3 and ETV5 act combinatorially to drive pluripotency progression, but the absence of both is not sufficient to prevent loss of ESC identity.

### Deletion of *Rbpj* Delays Naive State Exit

**Figure 2:**
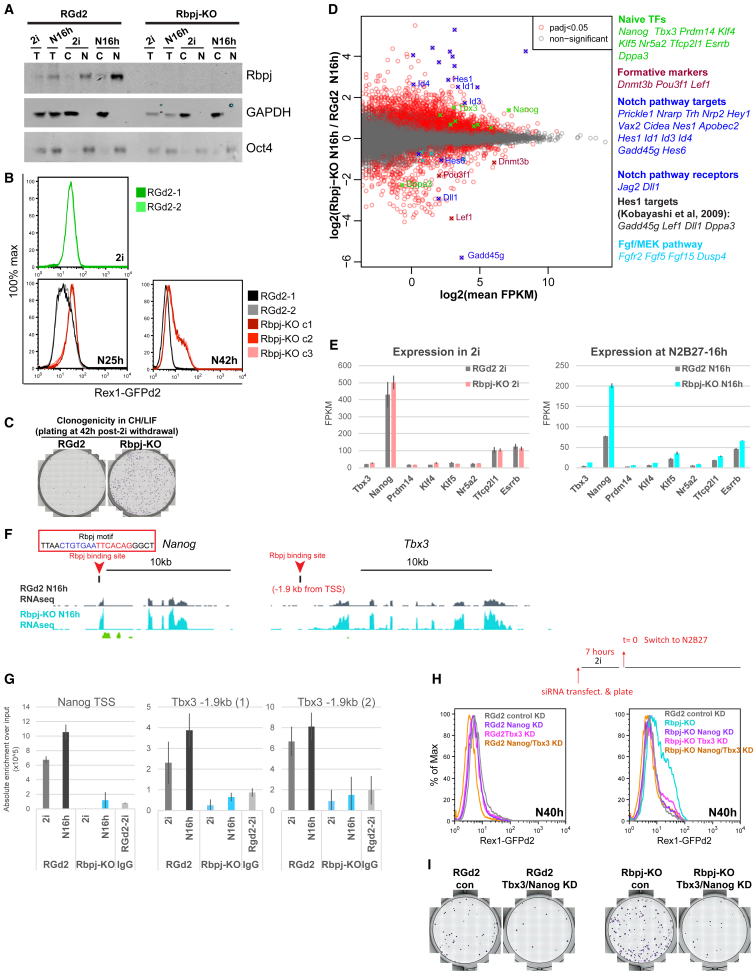
RBPJ Expression and Function (A) RBPJ western blot: C, cytoplasmic fraction; N, nuclear fraction; T, total cell lysate. Oct4 and GAPDH were used as loading controls for nuclear and cytoplasmic fractions, respectively. (B) GFP profiles of RGd2 and three clonal *Rbpj* mutant lines in N2B27 at 25 h (N25h) and 42 h (N42h) post-2i withdrawal. (C) Colony assay. (D) MA plot showing mean expression against fold change per gene in *Rbpj*-KO ESCs at 16 h post-2i withdrawal (N16h). Gene symbols and colored tags are shown for selected genes listed. (E) RNA-seq expression values for naive pluripotency factors in RGd2 and *Rbpj*-KO ESC in 2i and at N16h. Error bars show SD from biological replicates plated in parallel; 3 independent clonal lines for *Rbpj*-KO and 2 different lines for RGd2 (one parental and one clonal). (F) The University of California, Santa Cruz (UCSC) genome browser tracks for *Nanog* and *Tbx3* loci showing normalized RNA-seq read coverage for parental and *Rbpj*-KO ESCs at N16h. RBPJ binding sites are indicated with red arrowheads. The RBPJ-binding motif within the *Nanog* locus is highlighted. (G) ChIP-qPCR for binding sites shown in Figures 2F and S4E. Two primer sets were used for the *Tbx3* locus. y axis shows absolute enrichment normalized to input DNA for each sample. Error bars indicate SD from two ChIP replicates. (H) GFP profiles at 40 h after 2i withdrawal following a 7-h period of siRNA transfection. (I) Colony assay at 40 h after 2i withdrawal. See also Figures S2, S3, and S4 and Tables S1 and S2.

We re-inspected the candidate regulators to identify a factor that might complement ETV5 and TCF3 to enforce exit from naive pluripotency. The repressor RBPJ was detected in a haploid ESC mutagenesis screen (Leeb et al., 2014). RBPJ is expressed in the naive epiblast in the embryo (Figure S2C). RBPJ is nuclear localized in ESCs (Figure 2A), and RBPJ mRNA and protein are upregulated upon 2i withdrawal, a response that is enhanced in Etv5-KO and *Tcf3*-KO cells (Figures S2D–S2F). RBPJ is therefore a candidate complementary regulator, regulated by both ERK and GSK3, and acting through uncharacterized targets.

We inactivated *Rbpj* in RGd2 ESCs using CRISPR/Cas9. *Rbpj*-deficient ESCs adopted a more flattened colony morphology but showed no markers of differentiation (Figure S2G) and growth in 2i remained similar to parental ESCs (Figure S2H). Upon withdrawal from 2i, *Rbpj*-KO cells exhibit delayed downregulation of Rex1-GFPd2 and persistence of clonogenic ESCs at 42 h, consistent with siRNA results (Leeb et al., 2014; Figures 2B and 2C). Rex1 downregulation timing is restored upon expression of an *Rbpj* transgene, which also rescues domed colony morphology (Figures S2G and S2I).

RBPJ is best known for a role in the NOTCH pathway in which activated NOTCH intracellular domain induces a switch from repression to activation of target genes (Kopan and Ilagan, 2009). However, RBPJ can also regulate genes independently of NOTCH (Castel et al., 2013, Johnson and Macdonald, 2011). Absence of most known NOTCH transcriptional targets (Figure S2J), despite detectable expression of NOTCH ligands and pathway components (Figure S2K), suggests that the NOTCH pathway may not be significant during naive ESC transition. Nonetheless, to test whether NOTCH might be relevant to exit dynamics, we employed γ-secretase inhibitors to block production of NOTCH intracellular domain. We did not observe any effect on Rex1-GFP downregulation (Figure S2L). Thus, RBPJ may act purely as a repressor during naive state exit.

By RNA-seq, we found 405 upregulated and 705 downregulated genes in *Rbpj*-KO ESCs in 2i (p adj. ≤ 0.05; fragments per kilobase per million mapped reads [FPKM] ≥ 1; Table S1), with functions in multiple processes (Figures S3A and S3D). Because *Rbpj* deletion did not affect ESC self-renewal (Figure S2H), we focused on differential expression during transition. At 16 h after 2i withdrawal (N16h), 2,341 genes were up- and 355 downregulated (Table S1). The Kyoto Encyclopedia of Genes and Genomes (KEGG) pathway analysis shows enrichment in both 2i and N16h for cell adhesion, focal adhesion, and extracellular matrix (ECM)-receptor interactions (Figures S3A and S3B), reflected in expression of laminins, integrins, collagens, and cadherins (Figure S3C). This is in line with observations in *Rbpj* mutant fibroblasts (Castel et al., 2013) and likely explains the morphology of *Rbpj*-KO ESCs. Formative pluripotency markers *Lef1*, *Dnmt3b*, and *Pou3f1* fail to be upregulated (Figures 2D and S3E). Reduced expression of FGF/mitogen-activated protein kinase (MAPK) pathway components and NOTCH receptors is also evident. Among the top upregulated genes are targets of canonical NOTCH signaling known to be repressed by RBPJ, including *Id1*, *Id3*, *Id4*, and *Hes1* (Main et al., 2010, Meier-Stiegen et al., 2010; Figures 2D, S3E, and S4A). Repressed targets of Hes1 (Kobayashi et al., 2009) are among the top downregulated genes, consistent with increased Hes1 levels. ID factors and HES1 have previously been shown to impede ESC differentiation (Davies et al., 2013, Kobayashi and Kageyama, 2010, Ying et al., 2003, Zhou et al., 2013). Id3 is highly expressed in *Rbpj* mutants (Figure S4A). CRISPR/Cas9-mediated mutation of *Id3* in RGd2 ESCs slightly diminished GFP levels and appeared to accelerate downregulation. However, *Id3* knockout did not restore exit kinetics in *Rbpj*-KO ESCs (Figure S4B). We also mutated *Hes1* and found no effect (Figure S4C).

To widen the search for relevant targets, we examined pluripotency factor expression in *Rbpj* mutants and noted that *Tbx3* and *Nanog* are among the top 200 upregulated genes during transition (Figures 2D and 2F). A previous ChIP-seq study reported these genes among candidate RBPJ targets in F9 embryonal carcinoma cells (Lake et al., 2014). We employed ChIP-PCR to examine RBPJ binding at the reported *Nanog* and *Tbx3* sites and confirmed localization proximal to both genes (Figures 2F, 2G, and S4D). Furthermore, there is a palindromic RBPJ binding motif (Castel et al., 2013) within the binding site at the *Nanog* transcriptional start site (Figure 2F). We used siRNA to knock down *Nanog* and *Tbx3* in *Rbpj* mutants and found in both cases that exit kinetics and clonogenicity are almost fully restored (Figures 2H and 2I). We also tested the effect of these knockdowns in *Etv5* mutants (Figure S4E). NANOG depletion reverts the *Etv5*-KO exit delay phenotype, reflecting its general importance for sustaining naive pluripotency, whereas TBX3 siRNA has little or no effect, indicating that its role is specifically significant in the RBPJ context.

These data indicate that upregulation of RBPJ promotes extinction of naive pluripotency principally by extinguishing expression of *Nanog* and *Tbx3*. In addition, intersection of the ChIP-seq data with transcriptome data from Rbpj-KO cells identified 401 potential directly repressed genes (Figure S4F; Table S2), including other genes associated with ESC self-renewal, notably the LIF signal transducer STAT3. Significantly, only 8 of these candidates overlap with high-confidence TCF3 repressed targets (Figure S4G; Table S2; Martello et al., 2012).

### Elimination of *Etv5* and *Rbpj* Allows Self-Renewal Supported Only by GSK3 Inhibition

**Figure 3:**
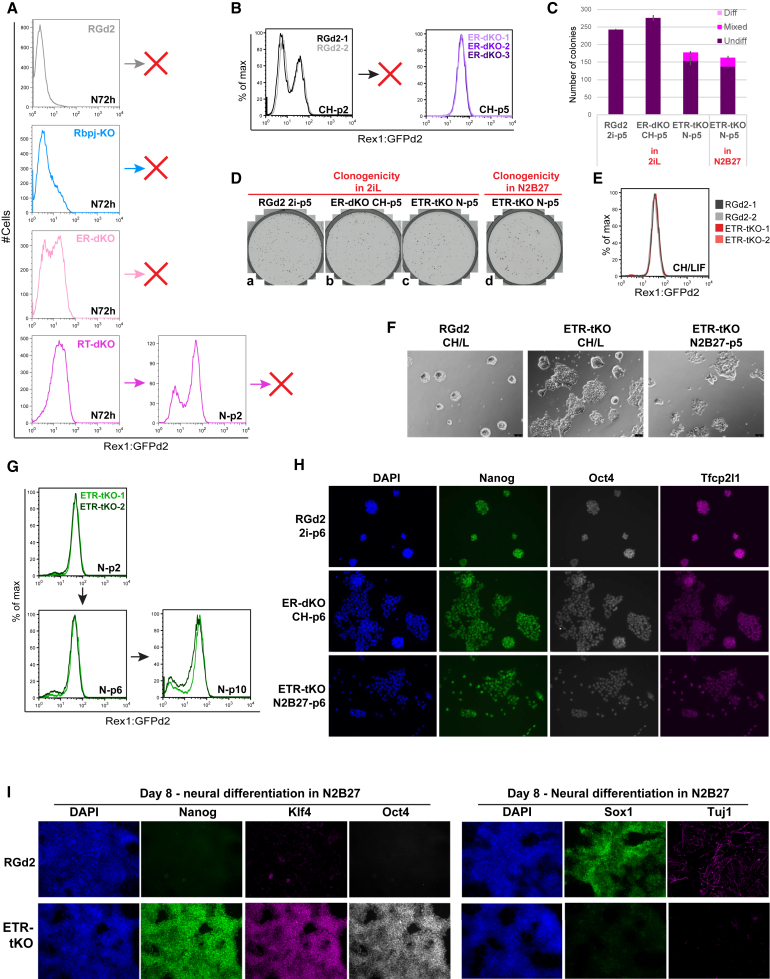
Dual- and Triple-Knockout Phenotypes (A) GFP profiles of RbpJ-KO (R-KO), Etv5/Rbpj-dKO (ER-dKO), and Rbpj/Tcf3-dKO (RT-dKO) ESCs at 72 h post-CH/LIF withdrawal (N72h) or at the end of passage 2 (p2). Red crosses indicate failure of replating upon passage. (B) Profiles of RGd2 and ER-dKO ESCs cultured in CHIRON (CH) only. (C) Clonogenicity in 2i/LIF or N2B27. Error bars show SD from 2 technical replicates. (D) Whole well images of colony formation in 2i/LIF or N2B27. (E) GFP profiles of ETR-tKO and RGd2 ESCs cultured in CH/LIF. (F) Phase contrast images. Scale bar represents 75 μM. (G) GFP profiles of ETR-tKO ESCs cultured in N2B27 only. (H) Immunofluorescent staining (IF) of RGd2 ESCs cultured in 2i, ER-dKO in CH, and ETR-tKO in N2B27 after 6 passages. (I) IF after 8 days of neural differentiation. (H and I) Images were taken using 20× (H) and 10× (I) objective. See also Figures S4 and S5.

In light of their independent regulation (Figures S2A–S2F), we created combined mutants for both *Etv5* and *Rbpj* (ER-dKO) and *Rbpj* and *Tcf3* (RT-dKO). Double mutants show a stronger delay phenotype, but undifferentiated ESCs cannot be expanded beyond two passages in N2B27 (Figure 3A). However, deficiency for *Etv5* and *Rbpj* is sufficient to sustain self-renewal in GSK3 inhibitor (CH) only. Parental ESCs cannot be propagated in these conditions beyond passage 2 (Figure 3B). Both single mutants can also be maintained in CH (Figure S4H) but expand slowly (Figure S4I), whereas ER-dKO cells proliferate similarly to parental RGd2 cells in 2i (Figure S4I) and remain uniformly GFP positive (Figure 3B). After 5 passages in CH, we assayed colony formation in 2i/LIF as a measure of naive ESC frequency. ER-dKO cells generated undifferentiated colonies with undiminished efficiency relative to RGd2 cells maintained in 2i (Figures 3C and 3D, a and b). Furthermore, they could also form colonies robustly in CH (Figure S4J), a property that is lost in parental ESCs after 2 passages. These results demonstrate a combined effect of *Etv5* and *Rbpj* deletion that enables self-renewal without MEK inhibition or LIF.

### Triple Deletion of *Etv5*, *Rbpj*, and *Tcf3* Renders ESC Self-Renewal Constitutive

We then generated triple knockouts for *Etv5*, *Rbpj*, and *Tcf3* (ETR-triple knockout [tKO]). Like *Rbpj*, *Tcf3*, and ER-dKO mutants, ETR-tKO cells were flattened but undifferentiated and uniformly GFP positive in CH/LIF (Figures 3E, 3F, and S5A). In contrast to other mutants, two independently generated ETR-KO clones maintained robust GFP expression in N2B27 (Figure 3G), expanding constantly although more slowly than in CH/LIF (Figures S5B and S5C). After 10 passages in N2B27, only a minor GFP-negative population emerged (Figure 3G). ETR-tKO cells passaged in N2B27 generated numerous alkaline-phosphatase-positive colonies on replating at clonal density in 2i/L and also in N2B27 only (Figures 3C and 3D, c and d). Immunostaining of ER-dKO cells in CH and ETR-tKO cells in N2B27 showed relatively homogeneous staining for OCT4 and for naive pluripotency factors NANOG and TFCP2L1 (Figure 3H). Under neural differentiation conditions, ETR-tKO cells maintained Nanog and Klf4 protein expression with no induction of neural markers SOX1 or TuJ1 (Figure 3I).

**Figure 4:**
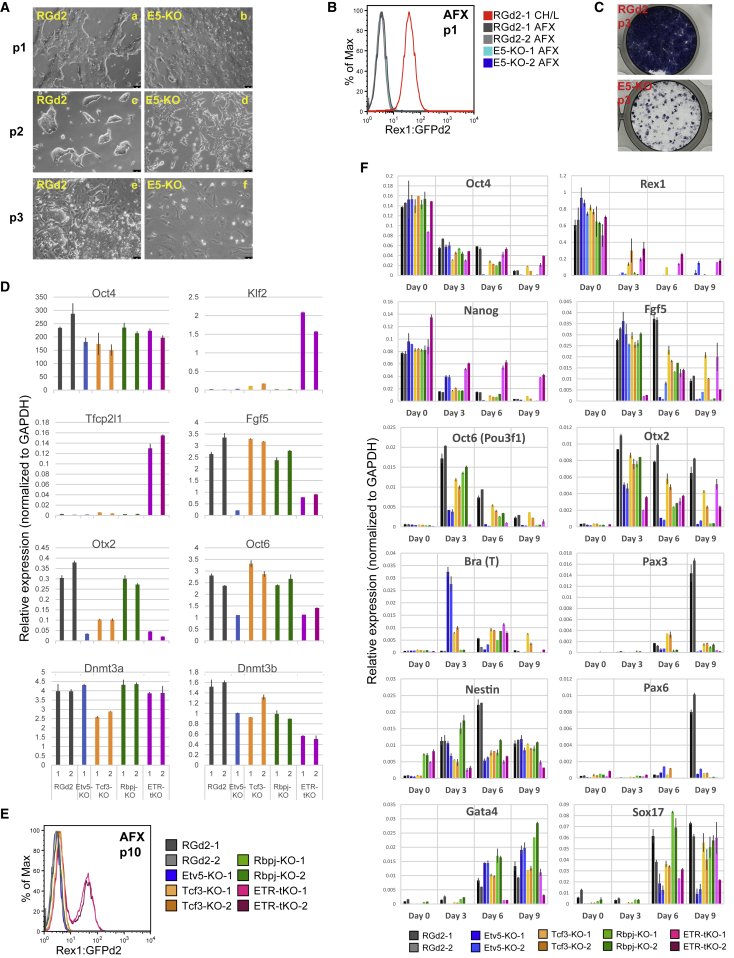
Transition Failure of Etv5 and Triple-Knockout ESCs (A) Phase contrast images of RGd2 and Etv5-KO ESCs during first three passages (p1–p3) in AFX taken using a 10× objective. Scale bars represent 75 μM. (B) GFP profiles at the end of p1. (C) Alkaline phosphatase staining at the end of p3. (D and E) qRT-PCR (D) and GFP (E) profiles after 10 passages in AFX. (F) qRT-PCR on embryoid bodies on days 3–9. Day 0 is starting ESCs in CH/LIF. Error bars in (D) and (F) show SD from 2 technical replicates for qPCR.

We examined whether conversion to primed EpiSC (Brons et al., 2007, Guo et al., 2009, Tesar et al., 2007) is impeded in the various mutants. Cells were transferred to medium containing activin, Fgf2, and Wnt pathway inhibitor XAV939 (Sumi et al., 2013), hereafter AFX. Over 3 passages, RGd2 lines converted into epithelial EpiSC with complete loss of GFP but retention of alkaline phosphatase (Figures 4A–4C). *Tcf3*-KO and *Rbpj*-KO mutants similarly converted efficiently to EpiSC. In contrast, *Etv5*-KO cells downregulated GFP (Figure 4B) but displayed distinct morphology by passage 2 (Figure 4A, c and d). By passage 3, cultures differentiated into fibroblast-like cells (Figure 4A, e and f) and contained only occasional patches of alkaline-phosphatase-positive cells (Figure 4C). Only one culture eventually yielded with EpiSC-like cells, and these cells deviated from the EpiSC state, with lower expression of *Pou3f1* and almost no *Fgf5* or *Otx2* (Figure 4D). In contrast to all the above, ETR-tKO mutants retained a significant proportion of GFP cells, even after 10 passages in AFX (Figure 4E). They maintained substantial expression of naive markers *Klf2* and *Tfcp2l1* with low expression of *Otx2*, *Pou3f1*, and *Fgf5* (Figure 4D).

We examined embryoid body formation in serum, an inductive system for multilineage differentiation (Doetschman et al., 1985). Single mutants downregulated naive markers but failed to upregulate the mesoderm marker PAX3 or neural marker PAX6 (Figure 4F). Endoderm markers were less affected, but this may reflect extraembryonic differentiation without formative transition (Smith, 2017). In ETR-tKO cells, upregulation of formative and lineage markers was severely diminished or delayed and expression of naive pluripotency markers persisted.

We investigated whether paracrine signaling might contribute to ETR-KO cell resistance to transition. We labeled ETR-tKO cells with the monomeric Kusabira Orange (mKO) reporter and set up mixed cultures with a minority (5%) of RGd2 test cells. GFP downregulation kinetics were unaltered (Figure S5D), demonstrating that the ETR-tKO phenotype is cell intrinsic.

Finally, we introduced a transgene for re-expression of *Etv5* and *Tcf3* in ETR-tKO mutants. Doxycycline-induced expression was lower than endogenous wild-type levels, but cells initiated downregulation of *Rex1* and *Nanog* and upregulation of *Pou3f1* (Figures S5E and S5F). After 3 days in N2B27 alone, NANOG protein was absent from a high proportion of Dox-treated cells, whereas it remained uniformly present in untreated cells (Figure S5G). This rescue experiment indicates that the transition delay phenotype is reversible and directly attributable to the mutated genes.

### Whole-Transcriptome Analysis of *Etv5*/*Rbpj* and *Etv5*/*Tcf3*/*Rbpj* Mutant ESCs

**Figure 5:**
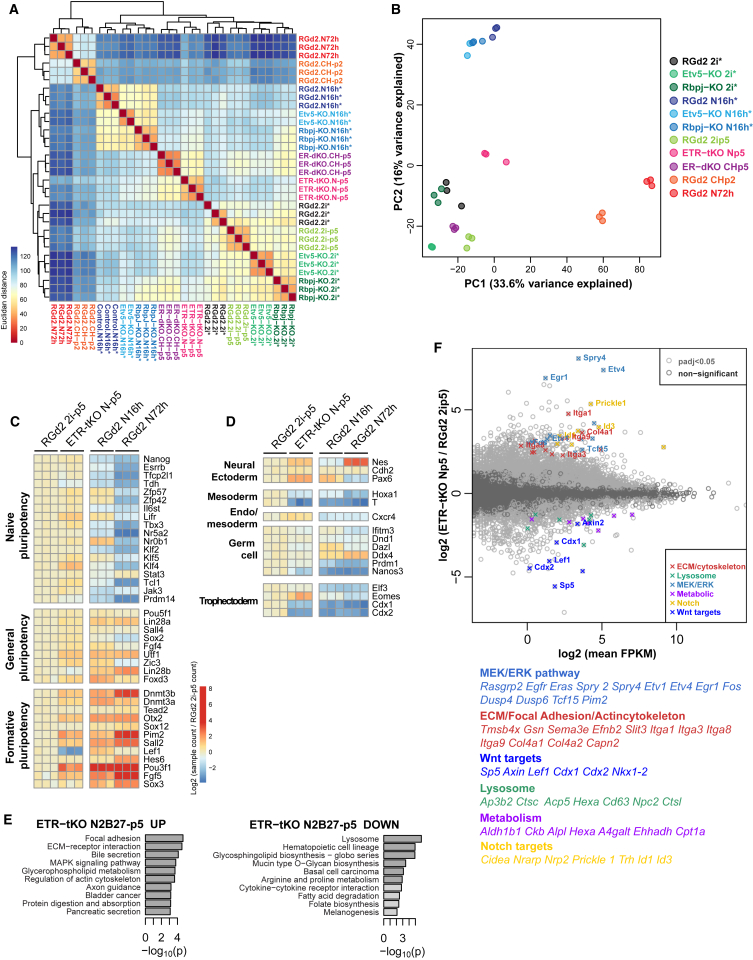
Transcriptome Analysis of Single and Combined Mutants (A and B) Hierarchical clustering (A) and PCA plot (B) based on normalized gene expression for all genes. ^∗^ denotes cells expanded in CH/LIF and switched to 2i for 48 h prior to sample conditions. (C and D) Heatmaps showing relative expression for pluripotency genes (C) and lineage markers (D). Values are shown as Log2 fold change of RNA-seq read counts relative to RGd2 2i-p5. Only the genes with a mean expression value of FPKM ≥ 1 in either RGd2 2i-p5 or ETR-tKO N-p5 samples were included. Genes were sorted by mean expression within each group. (E) KEGG pathway enrichment for differentially expressed genes. (F) MA plot showing mean expression against fold change per gene in ETR-KO cells cultured in N2B27 for 5 passages (ETR-tKO N-p5) versus RGd2 2i-p5 sample. Gene symbols are shown for selected genes listed below. See also Tables S2 and S3.

We performed RNA-seq (Table S3) and compared mutant cells with RGd2 cultures in 2i, N2B27 for 16 h or 72 h (N16h or N72h), or CH for 2 passages (CHp2). At 16 h, ESCs are poised for transition but will regenerate ESC colonies at high efficiency if restored to 2i/LIF (Kalkan et al., 2017). Hierarchical clustering (Figure 5A) divides N72h and CHp2 from other samples, consistent with having exited the ESC state. ETR-tKO cells cultured in N2B27 and ER-dKO cells in CH form a sub-cluster between 2i and N16h samples. Principal-component analysis (PCA) discriminates on PC1 samples before and after exit (Figure 5B), and PC2 separates 2i from transitional cells. ETR-tKO cells are close to 2i samples but displaced toward N16h.

Pluripotency factor profile is similar between ETR-tKO and naive ESC, with some modulation in levels (Figure 5C). Expression of some formative markers is detectable but at levels below those in N16h cells. Lineage markers are absent or very lowly expressed (Figure 5D; Tables S2 and S3). As in *Rbpj* single mutants, NOTCH targets, focal adhesion, and ECM genes are upregulated, along with actin cytoskeleton components (Figures 5E and 5F). MEK and ERK pathway components and targets are upregulated while expression of Wnt target genes is reduced, in line with absence of 2i (Figures 5E and 5F). Several metabolism- and lysosome-related genes are downregulated, which may relate to slower growth of ETR-tKO cells (Figure S5F).

These results establish that the naive pluripotency factor network is intact and the transition to formative pluripotency is barely initiated, both for *Etv5/Rbpj* mutants cultured in CH and for triple mutants in N2B27 only.

### Triple-Knockout Cells Colonize Chimeras but Do Not Convert to Post-implantation Epiblast

**Figure 6:**
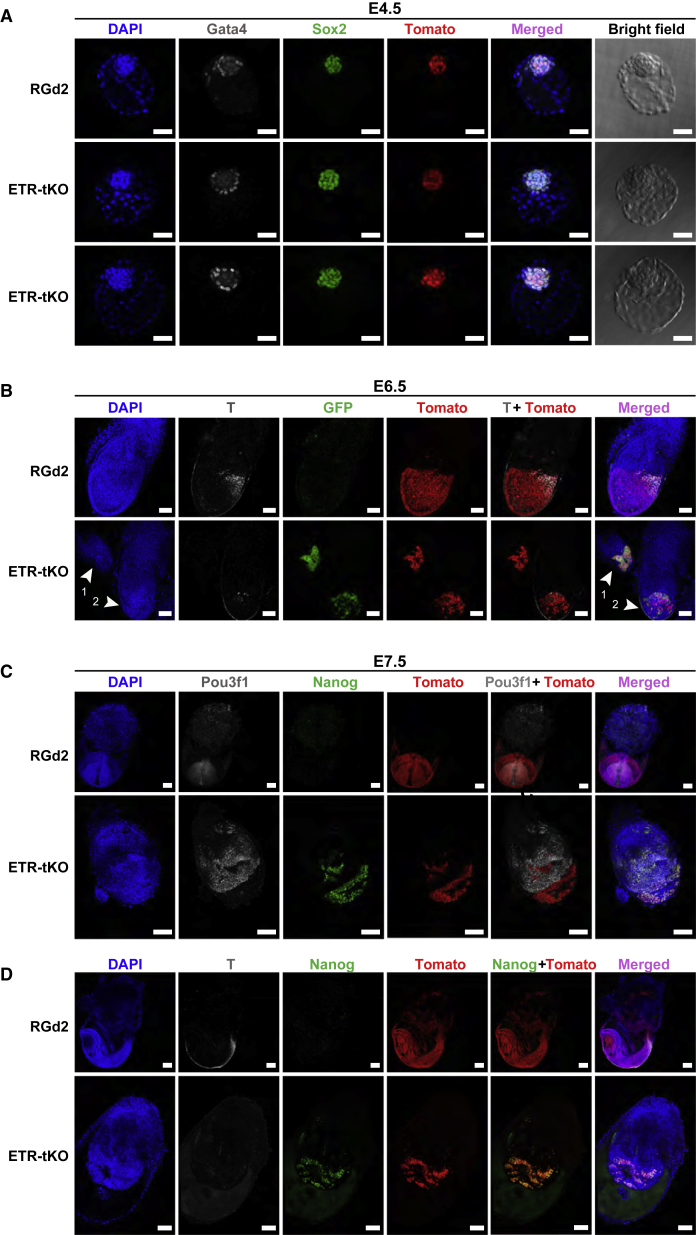
Chimera Contribution and Perturbation by Triple-Knockout ESCs Reporter fluorescence and whole-mount immunofluorescence staining on chimeric embryos obtained from RGd2 or ETR-tKO ESCs labeled with H2B-tdTomato (red). (A) *In vitro* matured blastocysts at E4.5 stained for Gata4 and Sox2. Scale bars represent 50 μm. (B) E6.5 embryos with T (Brachyury) staining and Rex1-GFP fluorescence. Arrowheads in the lower DAPI panel point to separate ETR-tKO chimeras. Scale bars represent 100 μm. (C) E7.5 embryos with Pou3f1 (Oct6) and Nanog staining. Scale bars represent 200 μm. (D) E7.5 T and Nanog staining. Scale bars represent 200 μm. Note the different magnifications for RGd2 and ETR-tKO chimeras in (C) and (D). See also Table S4.

We then examined whether ETR-tKO cells retain functional proximity to naive epiblast. We introduced a constitutive H2B*-*tdTomato reporter and performed injections into 8 cell embryos that were then cultured to the expanded blastocyst stage. In 9/9 blastocysts, mutant cells extensively colonized the epiblast, outnumbering the host cells similarly to parental RGd2 cells (Figure 6A). Contribution was confined to the Sox2-positive epiblast, with no cells detectable in primitive endoderm or trophoblast. Thus, ETR-tKO cells retain the ability of undifferentiated ESCs to survive, proliferate, and colonize the epiblast exclusively (Alexandrova et al., 2016).

We then examined behavior of ETR-tKO cells in post-implantation development following uterine transfer of injected embryos. Mutant cells were present in 20/20 embryos recovered at E6.5 and E7.5. Unlike RGd2 chimeras, which showed distribution of ESC progeny throughout the egg cylinder epiblast, most of the embryos injected with mutant cells had abnormal or rudimentary egg cylinders (see Table S4 for phenotypes and numbers). Mutant cells did not intermingle with host cells (Figures 6B–6D). They retained expression of Rex1-GFPd2 and of *Nanog* and failed to upregulate *T* (*brachyury*) or *Pou3f1*. In some cases, ETR-tKO contributions were large and extended beyond the embryonic-extraembryonic boundary (Table S4).

We conclude that triple mutant cells are unable to adopt identity of post-implantation epiblast and consequently cannot respond to inductive signals for germ layer specification. Their persistence in a naive-like state disrupts development of the host epiblast.

### ETV5 Regulates Network Components of Both Naive and Formative Pluripotency

**Figure 7:**
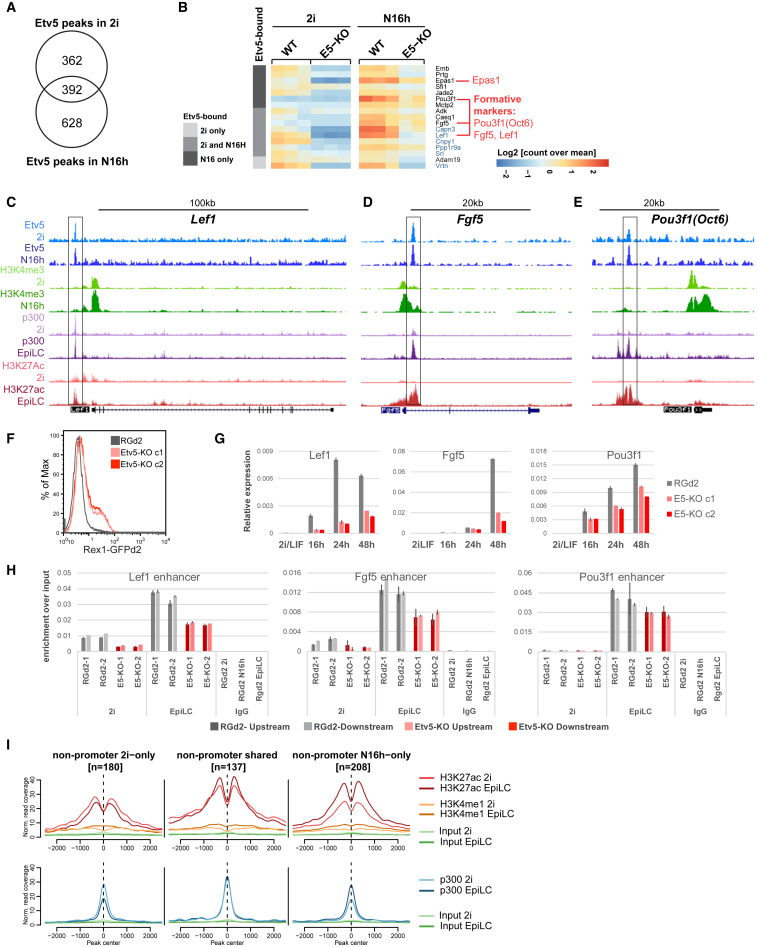
Etv5 Association with Transcriptionally Dynamic Genes (A) Numbers of ETV5 ChIP-seq peaks in 2i or at 16 h post-2i withdrawal (N16h). (B) Heatmap showing relative expression of downregulated genes in *Etv5* mutants (fold ≤ 0.66) at 16 h post-2i withdrawal (N16h) with proximal ETV5 binding. (C–E) UCSC Genome browser tracks of *Lef1* (C), *Fgf5* (D), and *Pou3fl* (E) loci showing normalized ChIP-seq read coverage for Etv5 and H3K4me3 (this study), p300, H3K27Ac, and H3K4me1 (Buecker et al., 2014). (F) GFP profiles of EpiLCs (48 h in Activin/Fgf2/KSR) generated from RGd2 ESCs or Etv5-KO ESCs (2 clonal lines). (G) qRT-PCR on time course samples during EpiLC formation. Error bars show SD from 2 wells of cells differentiated in parallel. (H) ChIP-qPCR showing H3K27Ac levels on upstream and downstream loci adjacent to the Etv5 peaks shown in (C)–(E). y axis shows absolute enrichment normalized to input DNA from each sample. ChIP was performed in duplicate (1 and 2) for each sample. Error bars show SD from 2 qPCR replicates. (I) Mean read coverage for p300, H3K27Ac, and H3Kme1 (ChIP-seq from Buecker et al., 2014) on “non-promoter”-associated ETV5-bound loci. Read depth is scaled to 1×. See also Figures S6 and S7 and Tables S5 and S6.

To illuminate how ETV5 regulates both self-renewal and transition, we performed RNA-seq and ChIP-seq in 2i and N16h. We identified 754 ChIP-seq peaks in 2i and 1,020 at N16h, with only 392 in common (Table S5; Figure 7A). Thus, there is a major change in ETV5 genome location early in the ESC transition process. As observed in other cell types for ETV5 and ETS factors in general (Hollenhorst et al., 2007, Zhang et al., 2017), ETV5 peaks were enriched at promoters (Figure S6A). RNA-seq in 2i revealed 77 downregulated genes (fold change [FC] ≤ 0.67) and only eight upregulated genes (FC ≥ 1.5) associated with ETV5 peaks (Figure S6B; Table S6), consistent with function of ΔN-ETV5 as a transcriptional activator. Targets include genes with potential roles in proliferation and maintenance of ESCs (Figure S6B): *Sall1* (Novo et al., 2016); *E2f2* (Wang and Baker, 2015); *Id3* (Ying et al., 2008); and most notably *Klf5*, which supports robust ESC proliferation (Ema et al., 2008). Activation of these genes may explain the contribution of ETV5 to ESC expansion. On the other hand, ETV5 binding is also detected at genes encoding transcription factors and epigenetic regulators associated with ESC transition or recovered in exit screens (Figure S6B). By priming transcription of these genes, ETV5 may prepare naive cells for rapid progression. Curiously, *Otx2* was upregulated in *Etv5*-KO cells in 2i, although this was not sustained during transition (Figures S6B and S7A).

At N16h, we found ETV5 binding proximal to 163 (FC ≥ 1.5) of 3,672 upregulated genes (p adj. ≤ 0.05; FPKM ≥ 1; Figure S6C; Table S6). These include transcription factors, components of H3K4 methyltransferase complex, negative regulators of Ras/ERK pathway, and transforming growth factor β (TGF-β) pathway members. *Id3* and some naive transcription factors are also represented. These data present the possibility that ETV5 might participate in transcriptional repression by an unknown mechanism and thereby contribute to shutting down the naive GRN. Intersection with TCF3 and candidate RBPJ-repressed targets showed limited overlap (Figure S6D).

Among 346 downregulated genes at 16 h, 16 are associated with proximal ETV5 binding (Figure 7B). These include genes in metabolic pathways and involved in calcium signaling. This is of note because metabolic resetting is an early feature of ESC transition (Fiorenzano et al., 2016, Kalkan et al., 2017, Zhou et al., 2012). Prominent formative pluripotency markers *Lef1*, *Fgf5*, and *Pou3f1* are also represented. ETV5 binds to enhancers associated with these three genes (Figures 7C–7E). The enhancers are activated in EpiLC (Buecker et al., 2014), a transient population obtained by plating ESCs in Activin/Fgf2/KSR for 48 h (Hayashi et al., 2011). We found that ETV5 remains bound in EpiLC (Figure S7B). *Etv5* mutants show impaired Rex1 downregulation (Figure 7F) and reduced expression of the three genes in EpiLC culture (Figure 7G). Furthermore, gain of H3K27Ac at the enhancers is diminished in mutants (Figure 7H), suggesting that ETV5 may promote H3K27 acetylation.

We used CRISPR/Cas9 to mutate *Pou3f1* and *Lef1* (Figure S7C). However, in neither single nor double mutants did we observe a delay in exit (Figure S7D). The marker profile of *Lef1*/*Pou3f1* double mutants at 48 h was also similar to parental RGd2 cells, although a modest reduction in Sox2, Sox3, and Fgf5 was apparent (Figure S7E). We also noted that ETV5 binds to the *Oct4* proximal enhancer that is required for expression in post-implantation epiblast (Figure S7F; Yeom et al., 1996) and found that *Oct4* expression was maintained at ESC levels during mutant cell conversion to EpiLC (Figure S7G). We then examined potential wider-reaching actions of ETV5. We partitioned non-promoter ETV5 peaks, which include enhancers, into 3 groups: 2i only; N16h only; and shared. Across these regions, we computed levels of chromatin marks associated with activated enhancers; H3K27Ac; H3K4me1; and p300, from Buecker et al. (2014; Figure 7I). We found that 2i-specific ETV5-bound regions lose H3K27Ac and p300 upon conversion to EpiLC, whereas across 208 loci that gain ETV5 at 16 h, there is a marked increase in H3K27Ac and p300 in EpiLC. A more modest gain in H3K27Ac is apparent across shared regions. Thus, ETV5 relocates from naive pluripotency-specific enhancers to formative/EpiLC enhancers upon 2i withdrawal. In contrast, promoters associated with ETV5 are largely devoid of p300 and show loss of H3K27Ac in EpiLC (Figure S7H). ETV5 binding is coincident with p300 at enhancers (Figures 7C–7E and 7I). This is of note because p300 has been shown to acetylate ETV1 and ETV4, increasing transactivation potential (Goel and Janknecht, 2003, Guo et al., 2011a), a mechanism likely also to operate for ETV5. Furthermore, depletion of p300 has an ESC transition delay phenotype (Leeb et al., 2014), consistent with p300 and ETV5 cooperating to commission formative enhancers.

## Discussion

This study demonstrates that elimination of two transcriptional repressors and one activator effectively prohibits mouse ESC progression to lineage competence. TCF3, RBPJ, and ETV5 serve complementary and partially overlapping functions in driving exit from the ESC ground state and initiation of formative pluripotency. Cells lacking all three factors are trapped in an ESC-like condition from which they can only rarely escape, even in the presence of strong differentiation stimuli.

Absence of TCF3 permits ESC propagation in MEK inhibitor alone (Wray et al., 2011), and combined deletion of ETV5 and RBPJ sustains self-renewal with GSK3 inhibition only. Triple-knockout cells are liberated from requirement for both inhibitors and exhibit constitutive self-renewal. These cells are stalled at a very early stage in transition. They retain uniform expression of naive pluripotency factors and high clonogenicity. Robust colonization of the naive epiblast demonstrates they remain functionally within the ESC compartment. However, ETR-tKO cells cannot advance from this state, even in the powerful inductive environment of the post-implantation embryo. These observations are consistent with evidence that ESCs may self-renew autonomously if differentiation is prevented.

TCF3 is well characterized as an ESC regulator (Cole et al., 2008, Guo et al., 2011b, Pereira et al., 2006, Wray et al., 2011). RBPJ by contrast has not previously been studied in this context. RBPJ is known to repress *Hes1* and *Id* genes, factors that can delay or reverse pluripotency progression in ESCs cultured in serum or bone morphogenetic protein (BMP) (Davies et al., 2013, Ying et al., 2003). In the peri-implantation embryo, RBPJ may counterbalance BMP to curtail persistence of naive epiblast. However, during defined *in vitro* transition, the critical contribution of upregulated RBPJ is to extinguish expression of naive factors, in particular *Nanog* and *Tbx3*, and prevent re-ignition of the naive GRN. Elimination of TBX3 may also be important to restrict potency for extraembryonic endoderm (Lu et al., 2011).

Combined deletion of *Etv4* and *Etv5* compromises proliferation and differentiation of ESCs (Akagi et al., 2015). We find that both phenotypes are primarily attributable to Etv5. This is in line with post-implantation lethality by E8.5 of ETV5 ETS domain deletion (Lu et al., 2009), whereas *Etv4*-null mice are viable (Arber et al., 2000, Laing et al., 2000). *Etv5* mutant mice that carry an N-terminal deletion are also viable (Chen et al., 2005), supporting functionality of ΔN-Etv5. ΔN-ETV5 may have altered signal sensitivity and partner interactions, which may be of relevance in cancers in which PEA3 family members are frequently mis-expressed (de Launoit et al., 2006, Hollenhorst et al., 2011a). ΔN-ETV5 supports ESC propagation when ERK signaling is inhibited, potentially via direct regulation of *Klf5*. When ERK is active in self-renewal conditions, ETV5 is dispensable, likely due to activation of an alternative ETS factor, such as ETV4 or GABPA.

During naive GRN collapse, ETV5 pivots from supporting naive ESC propagation to activating the formative pluripotency program. Absence of ETV5 derails installation of the formative GRN and also impedes exit from naive pluripotency. This phenotype is distinct from *Tcf3* and *Rbpj* mutants, in which there is a delay in exit but no major compromise in subsequent transition or formation of EpiSC. However, mutations in peri-implantation epiblast factors implicated in formative pluripotency, such as *Pou3f1* and *Lef1*, do not substantially delay naive state exit. Initial handover to the formative GRN may therefore be specifically dependent on ETV5. We surmise that, in the absence of ETV5, the network switch is not initiated and cells transiently retain, or revert to, naive status. Under influence of TCF3 and RBPJ, they eventually exit but then mostly succumb to miscellaneous differentiation or death, although there is some rescue by ETV4 or other pathways.

PEA3 factors are known as transcriptional activators (Oh et al., 2012). Interestingly, even in ground-state ESCs, ΔN-ETV5 may prime transcription of some early transition genes (Figure S6B). Upon 2i withdrawal, ETV5 occupies new genome locations, many associated with enhancers that become active during or after transition. ETV5 is phosphorylated by active ERK1/2 and is a probable target for acetylation by p300. These effects likely lead to the observed relocation whereby ETV5 can rapidly contribute to commissioning the formative GRN.

ETR-tKO cells retain core features of ESC identity but operationally appear nullipotent because they are unable to execute the formative transition. We conclude that timely and correct developmental progression from naive pluripotency is determined by three functions: TCF3 triggers dissolution of the naive GRN; RBPJ enforces exit by preventing reversion; and ETV5 commissions the successor formative GRN. Additional regulators contribute (Betschinger et al., 2013, Kalkan and Smith, 2014, Leeb et al., 2014, Li et al., 2018), but these three actions may be the major drivers. Speed and efficiency depend on temporal coordination, and it is striking that all three components are already present in naive ESCs, although levels and activity change during transition. It will be of interest to compare with mammals that have prolonged pluripotency progression, including primates (Nakamura et al., 2016, Smith, 2017). Future research will also reveal whether dissolution, enforcement, and initiation effects may commonly be combined to provide a triple lock for secure cell state transition.

## STAR★Methods

### Key Resources Table

**Table:** 

REAGENT or RESOURCE	SOURCE	IDENTIFIER
**Antibodies**		

pERK p44/42 MAPK (T202/T204) XP	Cell Signaling Technology	Cat#4370S
Etv5	Abcam	Cat#ab102010; RRID:AB_10711030
Flag M2	Sigma-Aldrich	Cat#F1804; RRID:AB_262044
Rbpj	Cell Signaling Technology	Cat#5442S; RRID:AB_10695407
Normal rabbit IgG	Cell Signaling Technology	Cat#2729S
Normal rabbit IgG	Abcam	Cat#ab171870
Normal mouse IgG	Santa Cruz	Cat#sc-2025
GAPDH	Sigma-Aldrich	Cat#G8795; RRID:AB_1078991
H3K27Ac	Active Motif	Cat#39135; RRID:AB_2614979
ERK	Cell Signaling Technology	Cat#9107
Pou3f1 (Oct6) C-20	Santa Cruz	Cat#sc-11661; RRID: AB_2268536
Lef1	Abcam	Cat#ab137872
GATA4	Santa Cruz	Cat#sc1237; RRID: AB_2108747
Sox2	eBioscience	Cat#14-9811-80; RRID: AB_11219070
Tuj1	R&D Systems	Cat#MAB1195
Sox1	Cell Signaling Technology	Cat#4194; RRID:AB_1904140
Klf4	R& D Systems	Cat#AF3158; RRID:AB_2130245
Nanog	eBioscience	Cat#14-5761-80; RRID:AB_763613
Tfcp2l1	R& D Systems	Cat#AF572; RRID:AB_2202564
H3K4me3	Diagenode	Cat#pAb-MEHAHS-024
GFP	ThermoFisher Scientific	Cat#A-11122; RRID:AB_221569
T (Brachyury)	R&D Systems	Cat#AF2085; RRID:AB_2200235
Oct4	Santa Cruz	Cat#sc-5279; RRID:AB_628051
b-tubulin	Abcam	Cat#ab6046; RRID:AB_2210370

**Chemicals, Peptides, and Recombinant Proteins**		

MEK inhibitor PD0325901	ABCR	Cat#AB 253775
GSK3 inhibitor CHIR99021	ABCR	Cat#AB 253776
Laminin	Millipore	Cat#CC095-5MG
Fibronectin	Millipore	Cat#FC010
ROCK inhibitor Y-27632	Merck Chemicals	Cat#688000-100MG
Tankyrase inhibitor XAV939	Cell Guidance Systems	Cat#SMS38-200
Activin A	Made in house	N/A
Fgf2	Made in house	N/A
LIF	Made in house	N/A
N2B27	Made in house	N/A
Accutase	Millipore	Cat#SCR005
Gelatin	Sigma-Aldrich	Cat#G1890
Γ-secretase inhibitor	Calbiochem	Cat#CAS 209984-56-5
Γ-secretase inhibitor DAPT	Merck Chemicals	Cat#565770
FuGENE HD transfection reagent	Promega	E2311

**Critical Commercial Assays**		

Alkaline Phosphatase Kit	Sigma-Aldrich	Cat#86R-1KT
NEPER Nuclear Cytoplasm Extraction Reagents	ThermoFisher Scientific	Cat#78833
Dynabeads Protein G	Thermofisher Scientific	Cat#10004D

**Deposited Data**		

Etv5 ChIP-seq (2i, N16h)	This study	GEO: GSE122338
H3K4me3 ChIP-seq (2i, N16h)	This study	GEO: GSE122338
RNA-seq of Etv5, Rbpj and Tcf3 single, double, and triple mutant ESCs	This study	GEO: GSE122338

**Experimental Models: Cell Lines**		

Rex1-GFPd2 (RGd2) ESC	Kalkan et al., 2017	N/A
Rex1-GFPd2 c1 (RGd2-2) ESC (clonal line)	This study	N/A
Etv5 knockout ESC (3 clonal lines)	This study	N/A
Rbpj knockout ESC (3 clonal lines)	This study	N/A
Tcf3-knockout ESC (2 clonal lines)	This study	N/A
Etv5/Tcf3 double knockout ESC (2 clonal lines)	This study	N/A
Rbpj/Tcf3 double knockout ESC (2 clonal lines)	This study	N/A
Etv5/Tcf3/Rbpj triple knock out ESC (3 clonal lines)	This study	N/A
Etv4 knockout ESC (3 clonal lines)	This study	N/A
Etv4/Etv5 knockout lines (1 clonal line)	This study	N/A
Hes1 knockout ESC (2 clonal lines)	This study	N/A
Id3 knockout ESC (2 clonal lines)	This study	N/A
Lef1 knockout ESC (2 clonal lines)	This study	N/A
Pou3f1 knockout ESC (2 clonal lines)	This study	N/A
Pou3f1/Lef1 double knockout ESC (2 clonal lines)	This study	N/A
RGd2/mKusabira Orange (mKO) ESC	This study	N/A
RGd2/ H2B-tdTomato ESC	This study	N/A
ETR-tKO/mKusabira Orange(mKO) ESC	This study	N/A
ETR-tKO/H2B-tdTomato ESC	This study	N/A
Etv5-3xFlag knockin ESCs (2 clonal lines)	This study	N/A
Etv5-KO/Etv5-3xFLAG rescue ESC	This study	N/A
Etv5-KO/ΔEtv5-3xFLAG rescue ESC	This study	N/A
Etv5-KO/mKusabira Orange (mKO)	This study	N/A
Rbpj-KO/Rbpj rescue ESC	This study	N/A
ETR-tKO/TetG/TRE3G-iEpT ESC	This study	N/A
ETR-tKO/TetG ESC	This study	N/A
Etv4/Etv5 double knockout ESC (2 lines; PE15-3, PE15-4)	Lu et al., 2009	N/A
Etv4/Etv5 dKO (PE3/PE4)/ mKusabira Orange ESC	This study	N/A
Etv4/Etv5 dKO (PE3/PE4)/ Etv5-3xFlag rescue ESC	This study	N/A
Etv4/Etv5 dKO (PE3/PE4)/ ΔEtv5-3xFLAG rescue ESC	This study	N/A
Wild type ESC	Lu et al., 2009	N/A

**Experimental Models: Organisms/Strains**		

*Mus musculus* females aged 6-10 weeks: CD1 strain was used to provide embryos and CBA/BL6 F1 animals were employed as transfer recipients for embryo chimeras.	N/A	N/A

**Oligonucleotides**		

Nanog siRNA, Mm_Nanog_3 FlexiTube siRNA	QIAGEN	Cat#SI01323357
Nanog siRNA, Mm_LOC100038891_2 FlexiTube siRNA	QIAGEN	Cat#SI04460869
Tbx3 pre-designed siRNA Assay ID: 223884	Thermofisher Scientific	Cat#AM16708
Tbx3 pre-designed siRNA Assay ID 223885	Thermofisher Scientific	Cat#AM16708
gRNAs Sequences	See Table S7 for sequences	N/A
Taqman and UPL gene expression assays	See Table S7 for oligo sequences and catalog numbers	N/A
ChIP-qPCR Primer Sequences	See Table S7 for sequences	N/A

**Recombinant DNA**		

TRE3G-Etv5-p2A-Tcf3-pGK-Hygo	This study	N/A
CAG-Etv5-3xFlag-pGK-Hygro	This study	N/A
CAG-ΔN-Etv5- 3xFlag-pGK-Hygro	This study	N/A
CAG-mKusabira Orange-pGK-Hygro	This study	N/A
CAG-Rbpj-pGK-Hygro	This study	N/A
CAG-H2B-tdTomato-IRES-Puromycin	This study	N/A
px459_SpCas9-2A-Puro	Addgene	#62988

**Software and Algorithms**		

Bowtie2	Langmead and Salzberg, 2012	N/A
MACS2	Zhang et al., 2008	N/A
DeepTools	Ramírez et al., 2016	https://doi.org/10.1093/nar/gkw257
Trim Galore!		https://www.bioinformatics.babraham.ac.uk/projects/trim_galore/
Bowtie2	Langmead and Salzberg, 2012	http://bowtie-bio.sourceforge.net/bowtie2/index.shtml
Samtools		http://samtools.sourceforge.net/
FeatureCounts	Liao et al., 2014	https://doi.org/10.1093/bioinformatics/btt656
R		https://www.R-project.org/
DESeq2		https://www.bioconductor.org/packages/release/bioc/html/DESeq2.html
Goseq		https://www.bioconductor.org/packages/release/bioc/html/goseq.html
Pheatmap		https://cran.r-project.org/web/packages/pheatmap/index.html

### Contact for Reagent and Resource Sharing

Further information and requests for resources and reagents should be directed to and will be fulfilled by the Lead Contact, Austin Smith, at austin.smith@cscr.cam.ac.uk

### Experimental Model and Subject Details

Mice used in these studies were adult females aged 6-10 weeks. The CD1 strain was used to provide embryos and CBA/BL6 F1 animals were employed as transfer recipients. Animals in the facility tested positive for *Helicobacter* but negative for other specific pathogens. Studies were performed in a UK Home Office designated facility in accordance with EU guidelines for the care and use of laboratory animals, and under authority of a UK Home Office project license. Use of animals in this project was approved by the Animal Welfare and Ethical Review Body for the University of Cambridge.

#### ES cell culture

Since Etv5-KO ESCs cannot be cultured long-term in the presence of PD, for consistency across experiments presented in this paper and with previous studies (Kalkan et al., 2017, Mulas et al., 2017), all cell lines were routinely cultured in CH/LIF then exchanged to 2i or 2iLIF for a total of 48hrs before the assay. For CH/LIF cultures, ES cells were plated at 1x 10^4^ cells cm-^2^ in CH/LIF on plates coated with 0.1% Gelatine (Sigma-Aldrich, G1890). Media was refreshed every other day and cells were passaged every 3 days. For passaging, cells were dissociated with Accutase (Millipore, SCR005) for 5 mins, 5-10x volume of wash buffer [DMEM/F12, 0.03% BSA Fraction V (Thermofisher)] was added, cells were spun and resuspended in fresh CH/LIF. Culture media used in the experiments consisted of N2B27 (made in house) supplemented with CH/LIF, 2i (CH/PD) or 2i/LIF at the following final concentrations: PD0325901 (PD), 1 μM; CHIR99021 (CH), 3 μM; LIF (prepared in house), 1:1000. To calculate growth rates, cell lines were plated at equal density, and counted at the end of each passage using a Vi-CELL Automated Cell Viability Analyzer (Beckman-Coultier).

#### Monolayer differentiation, clonogenicity assays and flow cytometry

For analyses of kinetics of exit from naive pluripotency, CH/LIF cultures were exchanged to 2i for 24hrs, dissociated with Accutase, plated in 2i at 1.5 × 10^4^ cells cm-^2^ and cultured for 24hrs prior to withdrawal of inhibitors. For flow cytometry, ESCs were dissociated using Accutase and diluted 1:5 in FACS buffer [PBS, 5% FBS]. ToPro-3 (Invitrogen) was added at a concentration of 0.05 nM to label membrane-compromised cells. Flow cytometry was performed on a Dako Cytomation CyAn ADP high-performance cytometer, using the same voltage settings for all experiments and results were analyzed with Flowjo. Representatives GFP profiles from at least 2 independent experiments are shown throughout the paper. For clonogenicity assays, cells were dissociated at 40-48h post-2i withdrawal and following resuspension in appropriate media, cells were plated at single cell density (∼500 cells/ 6-well) in 2i/LIF or CH/LIF on plates coated with 1% Laminin (Sigma, Cat. L2020) in duplicate. At day 6 alkaline phosphatase staining was performed using AP Kit (Sigma-Aldrich). Plates were scanned using Cell Celector (Aviso) and colonies were scored manually.

#### Notch Inhibition

RGd2 ES cells were treated with γ-secretase inhibitors DBZ (CAS 209984-56-5, Calbiochem) or DAPT (565770, Merck) at the concentrations shown in figure legends. DMSO was used as vehicle control.

#### Neural differentiation

Plates were coated with Laminin (∼10μg/ml in PBS) for at least 4 hours to overnight at 37°C. Laminin was aspirated and ESCs were plated directly in N2B27 onto laminin-coated plates at a density of 1.0x10^4^ cells/cm^2^. N2B27 was refreshed on day 2 and every day thereafter.

#### ES cell to EpiSC conversion and EpiSC culture

ES cells were plated in N2B27 supplemented with Activin A (20ng/ml), Fgf2 (12.5ng/ml) and XAV939 (1 μM) on Fibronectin-coated (Millipore, FC010) plates at a density of 1x10^4^ cells/ cm^2^. Media was refreshed on the 2^nd^ day and cells were passaged every 3 days using Accutase (Millipore, SF006). To enhance plating efficiency ROCK-inhibitor Y27632 (1mM) was included for the first 6-12 hours following plating and then removed.

#### Differentiation of ES cells in embryoid bodies (EB)

Single EBs per well were generated by sorting 1500 ES cells using a MoFLo Flow Sorter (Beckman Coultier) into a well of PrimeSurface96U plates (Sumitomo Bakelite) containing GMEM supplemented with GMEM, L-Glutamine (2mM), NEAA, Sodium Pyruvate (1mM), non-essential amino acids, β-Mercaptoethanol (100mM) (Life Technologies) and 15% FCS (Hyclone). Twelve EBs were pooled for assay.

#### Generation of Knock-Out ES cell lines using CRISPR-Cas9-mediated mutagenesis

gRNAs were cloned into PX459 (hSpCas9-2A-Puro) vector. Two gRNAs targeting different exons of a gene were co-transfected into Rex1-GFPd2 ESCs (Kalkan et al., 2017) using FuGENE HD (Promega). Transfected cells were selected using Puromycin (0.5-1 μg/ml) between 14h- 86h post-transfection. Clones were picked on day 9, expanded in CH/LIF and assayed by qRT-PCR to detect the genomic deletion. Details of gRNAs and qPCR primers are included in Table S7.

#### Genetic rescue of Etv5-KO, Rbpj-KO, ETR-tKO ES cell lines

cDNAs encoding *Tcf3*, *Rbpj* and long and short forms of *Etv5* were amplified from total cDNA of RGd2-N16h samples. *Rbpj* and *Etv5* isoforms were cloned into a PiggyBac vector containing a PGK-Hygromycin selection cassette and a CAG promoter to drive constitutive transgene expression. To generate stable “rescue” ES cell lines, 1x10^6^ Knock-Out ES cells were co-transfected with PiggyBac constructs (20ng) and PiggyBac transposase (400ng) using 1.5 μl FuGENE HD(Promega) for 14h in CH/LIF in one well of a 6-well dish. From 24h- post-transfection cells were cultured in Ch/LIF with Hygromycin (160μg/ml). For inducible expression of *Etv5* and *Tcf3* in ETR-tKO ESCs, an *Etv5*-p2A-*Tcf3* transgene was generated by PCR and cloned into a PiggyBac vector containing a PGK-Hygromycin selection cassette and a TRE3G promoter to drive Doxycycline-inducible transgene expression. 3x10^5^ ETR-tKO ES cells were co-transfected with this expression construct (50ng), a CAG-Tet3G-IRES-zeocin construct (50ng) and PiggyBac transposase (100ng), using 1μl FuGENE HD (Promega) for 14h in Ch/LIF in one well of a 6-well dish. From 24h-post-transfection cells were cultured with 20μg/ml Zeocin and 160μg/ml Hygromycin. Experiments were performed after at least 10 days of selection of stable transfectants with antibiotics. Doxycycline (100ng/ml) was added to culture media to induce transgene expression.

#### Generation of mKO- and H2B-TdTomato-labeled ESCs

1x10^6^ RGd2 and ETR-tKO ESCs were transfected as above with a Piggybac construct carrying CAG-driven monomeric Kusabira Orange (mKO) (20ng) and a PGK-Hygromycin selection cassette together with PiggyBac Transposase (400ng) and stable transfectants were selected with Hygromycin (160 ng/ml). For TdTomato labeling, 1x10^6^ ESCs were transfected with CAG-driven H2B-TdTomato-IRES-Puro construct using 10μl FuGene in one well of 6-well plate and selected with Puromycin (2 μg/ml).

#### siRNA transfection

RGd2 and *Rbpj*-KO ESCs cultured in CH/LIF were switched to 2i for 24hrs before siRNA transfection. Cells were dissociated and resuspended in 2i. 6x10^4^ cells were mixed with 700 μl 2i, 1μl Lipofectamine RNAiMax (Thermofisher) and 2 independent siRNAs for Tbx3 or Nanog or both, at a final concentration of 1.25nM each, and plated in a well of a 24-well tissue culture plate. AllStars negative control siRNA (QIAGEN) was used as control. After 7hrs, medium was replaced with N2B27 to initiate differentiation. Please see Table S7 for siRNA catalog numbers.

#### Immunofluorescence (IF) staining of ES cells

Cells were fixed for 10 min with 4%PFA at RT, followed by permeabilization and blocking in blocking buffer [PBS, 0.1% Triton X-100, 3% donkey serum] for 2hrs at RT. Cells were incubated with primary antibodies in blocking solution overnight at 4°C, using dilutions shown in Table S7. Alexa Fluor-conjugated donkey secondary antibodies (Molecular Probes) were used at 1:1000 dilution and were incubated with cells for 1hr at RT. Cells were washed with PBS/0.1% Triton X-100 three times for 5mins after primary and secondary antibody incubations. Images were obtained using a Leica 4000B standard fluorescent microscope using a 10x or 20x objective as indicated in the figure legends.

#### RNA extraction, cDNA synthesis and qPCR

Total RNA was isolated using Relia Prep RNA Miniprep System (Promega). cDNA was synthesized using GoScript Reverse Transcriptase System (Promega) and oligo-dT primers. qRT-PCR was performed with TaqMan Gene Expression (Thermo Scientific) or Universal Probe Library (Roche) assays. Expression levels were normalized to GAPDH for all analyses, except for Figure 1C for which TBP was used. Please see Table S7 for details.

#### Western blot and sub-cellular fractionation of total cell lysate

To obtain total cell lysate cells were lysed in 1xPBS supplemented with 1%Triton X-100, 0.1%SDS, protease and protein inhibitors (Roche) and sonicated briefly in the Bioruptor (Diagenode) to shear the gDNA. Extraction of nuclear and cytoplasmic fractions was performed with NEPER Nuclear and Cytoplasm Extraction Reagents (ThermoFisher Scientific). Blots were blocked with blocking solution (1xPBS, 1%Triton X-100, 5% skimmed milk) for 2hrs at RT, followed by incubation with primary antibodies for 2hrs at RT or overnight at 4°C. Primary antibodies and dilutions are listed in Table S6. For detection IRDye secondary antibodies (Li-Cor) were used at 1:2000- 1:5000 dilution and signal intensities were quantified by Odessey (Li-Cor). Antibodies and primer/probes sets are listed in Table S6.

#### Chromatin immunoprecipitation (ChIP) and ChIP-seq library preparation

ESCs were dissociated with Accutase, washed with culture medium (10x volume) and resuspended in fresh culture medium at 5x10^6^ cells per ml. To cross-link chromatin, for 1ml of cell suspension 100μl of Fix Solution [0.1M NaCl, 1mM EDTA, 0.5mM EGTA, 50mM HEPES pH 7.5, 11% Formaldehyde] was added, and cells were rotated for 10min at RT. To neutralize the formaldehyde, 157μl 1M Glycine was added, and cells were rotated for 5min at RT, followed by spin at 1600 g for 5min. Cells were then washed with 1ml ice-cold PBS/BSA 0.03% and spun, repeating 3 times. Protease /Phosphatase inhibitors (Roche) were added in the last wash. Cells were either frozen at −80°C at this point or processed immediately. To obtain nuclear lysates, pellets from 5x10^6^ fixed cells were resuspended in ice-cold 1ml buffer LB1 [50mM HEPES pH 7.5, 140mM NaCl, 1mM EDTA, 10% glycerol, 0.5% NP40, 0.25% Triton X-100], rotated for 10min at 4°C, spun at 1600 g for 5min, resuspended in ice-cold 1ml buffer LB2 [10mM Tris pH 8.0, 200mM NaCl, 1mM EDTA, 0.5mM EGTA], rotated for 10min at 4°C, spun at 1600 g for 5min and resuspended in 140μl shearing buffer [1% SDS, 10mM EDTA, 50mM Tris pH 8.0]. Nuclear Lysates were sonicated with Bioruptor (Diagenode) at High setting, for 26 cycles (30sec ON / 30sec OFF) to obtain DNA fragments with an average size of 300bp. Lysates were spun in a microcentrifuge at 8°C at maximum setting for 10mins to remove debris. 130 μl of supernanatant (equivalent of approximately 5x10^6^ cells) was diluted in 1300 μl 11x dilution buffer [50mM Tris pH 8.0, 167mM NaCl, 1.1% Triton X-100, 0.11% Na-Deoxycholate]. Lysates were frozen at −80°C at this point or processed immediately. 1430 μl diluted nuclear lysate (equivalent of approximately 5x10^6^ cells) was pre-cleared by incubating with 2-4μg of isogenic normal IgG and 25μl Protein G Dynabeads (Invitrogen, 1004D) on a rotator at 4°C for 2 hr. Supernatants were then incubated with the appropriate ChIP antibody (see Table S6 for dilutions) on a rotator overnight at 4°C, followed by incubation with 30μl Protein G Dynabeads for 1h at 4°C. To remove unbound chromatin and unspecific interactions, beads were washed two times with Wash Buffer 1 [50mM Tris pH 8.0, 0.1% SDS, 0.1% Na-Deoxycholate, 1% Triton X-100, 150mM NaCl, 1mM EDTA, 0.5mM EGTA], one time with Wash Buffer 2 [50mM Tris pH 8.0, 0.1% SDS, 0.1% Na-Deoxycholate, 1% Triton X-100, 500mM NaCl, 1mM EDTA, 0.5mM EGTA], one time with Wash Buffer 3 [50mM Tris pH 8.0, 250mM LiCl, 0.5% Na-Deoxycholate, 0.5% NP-40, 1mM EDTA, 0.5mM EGTA] and two times with Wash Buffer 4 [50mM Tris pH 8.0, 10mM EDTA, 5mM EGTA]. Each wash was performed for 5mins on a rotator using ice-cold buffers and protease/phosphatase inhibitors (Roche). Chromatin-antibody complexes were eluted by incubating in 125μl of Elution Buffer [1% SDS, 0.1M NaHCO3] on a shaker block at 37°C for 15mins, repeating twice. Elutions were pooled and DNA was purified using QIAGEN Minelute PCR purification kit.

The following antibodies and cell numbers were used for each ChIP replicate: H3K4me3 ChIP, 3x10^6^ cells, 3 μg rabbit H3k4me3 antibody (Diagenode pAb-MEHAHS-024, A1-010); H3K27Ac ChIP, 3x10^6^ cells, 2 μg rabbit H3K27Ac antibody (Active Motif, 39159); RBPJ ChIP, 5x10^6^, 4.5 μL polyclonal rabbit RBPJ antibody (Cell Signaling Technology, 5442S), Etv5-3xFlag ChIP; 5x10^6^ cells, 3 μg anti-Flag M2 antibody (Sigma-Aldrich). For H3K27Ac and RbpJ ChIP, 2μg normal rabbit IgG (Abcam,ab171870) was used for pre-clearing step and a different batch of rabbit IgG (Cell Signaling Technology, 2729S) was used for negative control samples. Etv5-Flag ChIP was performed on lysates obtained from two independently derived clonal ES cell lines (Etv5-Flag-KI-20 and Etv5-Flag-KI-32) that carry a 3xFlag epitope knocked in to the C-terminal end of the endogenous *Etv5* coding sequence just before the stop codon. Parental RGd2 ES cells served as negative control. ChIP-Seq libraries were generated using NEB Next ChIP-Seq Library Prep Master Mix Set for Illumina (NEB).

#### ChIP-seq analysis

Sequencing reads were mapped to the mm10 mouse reference genome using Bowtie2 (Langmead and Salzberg, 2012), converted to a density plot and displayed as UCSC genome browser custom tracks. ETV5 peaks were called over RGd2 parental line controls using MACS2 software (Zhang et al., 2008). Mapped reads were converted to density plots and displayed as UCSC genome browser custom tracks. Only the peaks called in both biological replicates (p < 10-4) were selected for further analyses. Peaks overlapping a promoter (1000+/− RefSeqGene TSS coordinates) by at least 1bp, or a gene body was assigned to only that gene. The rest of the peaks (intergenic peaks) were assigned to the nearest genes within 50Kb. To identify potential direct targets of Etv5, the peaks were intersected with genes that show a fold change of ≥ 1.5 in the UP direction and ≤ 0.66 in the DOWN direction in Etv5-KO ESCs over parental RGD2 ESCs.

H3K27ac, H3K4me1, p300 and input data from Buecker et al., 2014 with accession number GSE56138 were downloaded from the NCBI Gene Expression Omnibus. The reads were aligned to the mm10 reference genome using bowtie (-y -m 1–best–strata–nomaqround) and converted to bigwig using deeptools 6(Ramírez et al., 2016) bamCoverage (–extendReads 200–binSize 1–normalizeTo1x 2150570000). The mean signal at the ETV5 peaks was extracted using Deeptools computeMatrix using the ETV5 peak centers as reference points.

#### Transcriptome sequencing (RNA-seq)

RNA-seq was performed in triplicates per condition, including three independently derived clonal lines per genetic knock-out, and two RGd2 lines as wild-type controls. Exception is RGd2-N16h samples for which only 2 replicates were sequenced, as one was lost during library preparation. RGd2-2 is a clonal line derived from the parental RGd2 line (RGd2-1). Total RNA was extracted with ReliaPrep RNA Miniprep System (Promega) and was processed with Ribo-Zero capture probes (Illumina). Libraries were produced using NEXTflex Rapid Directional qRNA-Seq Kit (Bioo Scientific). Libraries were sequenced in the Illumina platform in paired-end mode.

#### RNA-seq analysis

Illumina sequencing adapters were removed using Trim Galore! and reads shorter than 20 nt were discarded. The reads were aligned to the mouse reference genome (GRCm38/mm10) with ERCC spike-ins appended to it. The alignment was done using TopHat2 and Gencode (release M14) gene models were used as a guide. Read counts per gene were calculated using featureCounts requiring strand-specific, primary and unique matches. Normalization and statistical analysis of the resulting counts table was done using the R Bioconductor package DESeq2 using normalization factors based on the spike-in counts. Gene counts were converted to fragments per kilobase per million mapped reads (FPKM) and then log2-transformed for visualization in heatmaps and MA-plots. A significance threshold of padj < 0.05 and FPKM ≥ 1 was used to define differentially expressed genes.

##### KEGG pathway analysis

Enriched KEGG pathways were identified using the ‘goseq’ package from R Bioconductor. Only expressed genes with mean FPKM ≥ 1 were considered. Differentially expressed genes with padj < 0.05 and log2 fold change > 0.5 or < −0.5 were compared with all expressed genes. False discovery rate was calculated to correct for multiple testing using the ‘p.adjust’ function in R.

##### Cluster analysis and PCA

Regularized log-transformed counts were calculated using DESeq2 and used for sample clustering and PCA. Hierarchical clustering was done using the ‘pheatmap’ package in R with sample distances calculated by the ‘dist’ function. PCA was done by the ‘prcomp’ function without scaling.

### Data and Software Availability

The accession number for ChIP-seq and RNA-seq data reported in this study is GEO: GSE122338.

#### Chimera production

Pregnant females were killed on day 2.5 post coitum (E2.5) by cervical dislocation. Oviduct and uterus were dissected, and flushed with M2 media (Sigma, M7167) using Leica M165C microscope system for better visualization. Embryos were collected in M2 media prior to microinjection. E2.5 embryos were transferred into M2 media, covered with a layer of mineral oil (Sigma, M8410). Embryos were visualized using an Olympus microscope system and an Olympus 40x LWD Plan APO 0.6 NA air objective. ESC were loaded into a microinjection pipette and injected into the perivitelline space of 8-cell embryos using Hamilton Thorne XYClone microinjection system (Hamilton Thorne). Eight cells were transferred into each embryo. Injected embryos were cultured for 2 days in Blast™ media (Origio, 8306001A) at 37°C, 20% O_2_ and 7% CO_2_. For post-implantation analyses, embryos were transferred one day after injections into oviducts of pseudopregnant females. Contribution was characterized at E4.5, E6.5 and E7.5.

#### Immunofluorescence staining of embryos

For immunofluorescence analysis of cultured pre-implantation stage embryos, zona pellucidae were removed using tyrode acid solution (pH 2.5). Embryos were fixed with 4% w/v paraformaldehyde (PFA) (Sigma, P6148) in PBS for 15 minutes at room temperature (RT). Samples were washed three times with PBS, supplemented with 3 mg/ml poly(vinylpyrrolidinone) (PVP) (Sigma, P0930) (PBS/PVP). For permeabilization, embryos were incubated for 30 minutes in 0.25% Triton X-100 (Sigma-Aldrich, T8787) diluted in PBS/PVP. Embryos were incubated for 15 minutes in 2% donkey serum, 0.25% bovine serum albumin (BSA) (Sigma, 1076192), 0.01% Tween 20 (Sigma Aldrich, P2287) in PBS, followed by overnight incubation in primary antibodies diluted in blocking buffer (Table S6). On the next day embryos were washed three times for 15 minutes in blocking buffer before incubation for 1h in secondary antibodies diluted in blocking buffer. Afterward, embryos were washed three times for 15 minutes in blocking buffer with or without DAPI.

Dissected post*-*implantation stage embryos were fixed for 1 hour in 4% PFA. Embryos were washed three times 15 minutes in PBS/PVP. For permeabilization, embryos were incubated for 1 hour in PBS containing 5% DMSO (Santa Cruz, sc-358801), 0.5% Triton X-100, 0.1% BSA and 0.01% Tween20 at 4°C. Embryos were blocked overnight at 4°C in humidified environment in permeabilization buffer, containing 2% donkey serum. On the next day embryos were incubated overnight at 4°C in primary antibodies (Table S7) in blocking buffer. Embryos were washed 3 times for 2 hours in blocking buffer, before incubation overnight in secondary antibodies diluted in blocking buffer. Lastly, embryos were washed three times for 2 hours in blocking buffer with or without DAPI.

For embryo mounting, samples were taken through a series of 25%, 50%, 75% and 100% vectashield (Vector Laboratories, H-1000) diluted in PBS. Embryos were mounted in a drop of vectashield, surrounded by drops of Vaseline as a spacer for the coverslip, to immobilise embryos. Coverslips were sealed using nail varnish. Finally, slides were stored at −20°C prior to imaging.

Embryos were imaged using a Leica TCS SP5 confocal microscope. Image acquisition was performed with a 20x 0.7NA air objective. For illumination, a 405, 488, 561 and 647 nm lasers and Leica application suite was used. Images were processed with Fiji software.

## Supplemental Information

Document S1. Figures S1–S7 and Table S4

Table S1. Differentially Expressed Genes in *Rbpj* Mutants and Intersection with Genes Repressed by RBPJ and TCF3, Related to Figures 2 and S4

Table S2. Expression of All Genes, Related to Figure 5

Table S3. Differentially Expressed Genes in ETR-KO Np5, Related to Figure 5

Table S5. ETV5-Associated Genomic Loci, Related to Figure 7

Table S6. Differentially Expressed Genes in *Etv5* Mutants and Intersection with Genes Regulated by ETV5, RBPJ, and TCF3, Related to Figures 7 and S6

Table S7. Reagents, Related to STAR Methods

Document S2. Article plus Supplemental Information
