# Supplementary material for: Complementary Activity of ETV5, RBPJ, and TCF3 Drives Formative Transition from Naive Pluripotency
Source: Cell Stem Cell. 2019 May 2;24(5):785–801.e7. doi: 10.1016/j.stem.2019.03.017 (PMC6509416; doi:10.1016/j.stem.2019.03.017)
Supplement: Document S1. Figures S1–S7 and Table S4 [file mmc1.pdf]

Cell Stem Cell, Volume *24*

Supplemental Information

Complementary Activity of ETV5, RBPJ, and TCF3 Drives Formative Transition from Naive Pluripotency

Tüzer Kalkan, Susanne Bornelöv, Carla Mulas, Evangelia Diamanti, Tim Lohoff, Meryem Ralser, Sjors Middelkamp, Patrick Lombard, Jennifer Nichols, and Austin Smith

Supplemental Figure Legends

Figure S1. ETV4/5 expression and function (Related to Figure 1)
(A) RNA-seq (data from Kalkan et al, 2017) and H3K4me3 ChIP-seq (this study) genome tracks for *Etv5*.
(B) Western blot with polyclonal ETV5 antibody for detection of endogenous ETV5 in RGd2 ESC. 2i cultures (0h) were switched to fresh 2i (2i), N2B27 (N) or single inhibitors (CH) or (PD) for 16 hours. Cells were transfected with negative control siRNA or siRNA against Etv5.
(**C, D)** Relative growth rates of: wildtype and *Etv4/5*-dKO ESC from Lu et al, 2009; RGd2 and *Etv5*-KO ESC lines. Cell numbers are normalized to WT for panel C, and to parental RGd2-1 ESC for panel D. Error bars indicate standard deviation (SD) from 2 wells cultured in parallel.
(E) Restoration of Rex1-GFPd2 downregulation kinetics in two *Etv5*-KO clonal ESC lines carrying stable transgenes encoding ΔN and canonical isoforms of ETV5.
(F) RT-qPCR for ETV4 in 2i and at indicated hours after 2i withdrawal. Error bars indicate SD from two wells cultured in parallel.

Figure S2. RBPJ expression and function (Related to Figures 1 and 2)
(A, B) TCF3 transcript levels in RGd2 and *Etv5*-KO ESCs in 2i and at hours (h) post-2i withdrawal.
(C-F) RBPJ transcript levels in: the early mouse embryo; RGd2 and Etv5-KO ESCs in 2i and hours (h) after 2i withdrawal; RGd2 and mutant ESCs cultured in CH/L and 28h after CH/LIF withdrawal. Error bars indicate SD from 2 or 3 biological replicates for RNA-seq, or from 2 wells cultured in parallel for RT-qPCR.
(G) Phase contrast images of parental, *Rbpj* KO and transgene rescued ESC; MT=empty vector, Rbpj tg=transgene encoding RBPJ. Scale bar= 0.75 μm
(H) Growth rates of two different RGd2 and *Rbpj*-KO ESC lines. All cell numbers were normalized to RGd2-1 ESC line.
(I) Restoration of Rex1-GFPd2 downregulation in *Rbpj*-KO ESC line stably expressing *Rbpj* transgene.
(J, K) Transcript levels of Notch target genes, and Notch pathway components.
(L) GFP profiles of RGd2 ESCs treated with Notch inhibitors (iNotch) or DMSO: (1) before 2i withdrawal; (2) after 2i withdrawal; (3) before and after 2i withdrawal.

Figure S3. RBPJ expression and function (Related to Figure 2)
(A, B) KEGG pathway enrichment for differentially expressed genes in *Rbpj* mutants vs. parental RGd2 ESC in 2i, and in N16h.
(C) Relative expression of genes associated with ECM and cell adhesion identified by KEGG pathway enrichment analysis.
(D) MA-plot showing mean expression against fold change per gene in *Rbpj*-KO ESC in 2i. Gene symbols are shown for indicated genes.
(E) Heatmaps showing relative expression of TOP 50 differentially expressed genes with mean FPKM≥1 in *Rbpj*-KO N16h for UP genes, and FPKM≥1 in RGd2 N16h for DOWN genes. Genes were ordered according to fold change of mean RNA-seq count in *Rbpj*-KO N16h over RGd2 N16h.

Figure S4. RBPJ expression and function (Related to Figures 2 and 3)
(A) Expression of HES1 and ID factors in *Rbpj* mutants. Error bars show SD for 3 independent clonal lines for *Rbpj*-KO, and 2 different lines for RGd2 (one parental, one clonal) cultured in parallel.
(B, C) Rex1-GFPd2 profiles of Id3 knock-out, and *Hes1* knock-out ESC. Data are shown for independent clonal lines cultured in parallel.
(D) ChIP-qPCR for RBPJ-bound loci identified by Lake et al, 2014. *Hes1* TSS and gene desert sites were used as positive and negative control regions, respectively. *Rbpj*-KO ESCs were used as negative control. Values were normalized to input DNA. Asterisks mark enrichment in *Nanog* and *Tbx3* promoter proximal sites. Error bars indicate SD from 2 qPCR replicates.
(E) GFP profiles at 29h after 2i withdrawal following a 7-hour period of siRNA transfection.
(F) Venn diagram showing the overlap of upregulated genes in *Rbpj*-KO ESCs at 16h post-2i withdrawal from RNA-seq (this study) and RBPJ-associated genes from published ChIP-seq dataset. Only genes that show a fold change of ≥1.5 increase in *Rbpj*-KO over RGd2 are considered. Pluripotency regulators are shown in boxes and naïve pluripotency transcription factors are in bold.
(G) Venn diagram showing intersection of potential targets repressed by RBPJ and TCF3.
(H) GFP profiles of parental RGd2 ESC, *Etv5/Rbpj* single and double mutants in CHIR99021 (CH) only. Crossed box indicates failure of expansion after passage 2 (p2).
(I) Growth rates of RGd2 ES cells in 2i, *Etv5/Rbpj* single and double mutants in CH. Error bars indicate SD from two independently-derived lines cultured in parallel.
(J) Colony formation by RGd2 and ER-dKO ES cells in CH or CH/LIF after culture for 2 passages (p2) in CH only. Zoomed-in images show larger colonies from ER-KO ESCs.

Figure S5. Dual and triple knock out phenotypes (Related to Figure 3)
(A) Bright field images of mutant lines taken using a 20x objective.
(B, C) Relative growth rates of indicated ESC over 5 passages in CH/L**,** and of RGd2 in 2i or ETR-tKO in N2B27. Independently derived ESC lines are numbered (1-3).
(D) Flow cytometry profiles **(a, c, e)** of mixed cultures comprised of unlabelled RGd2 ESCs and mKO-labelled RGd2 or mKO-labelled ETR-KO ESCs**,** and corresponding GFP profiles **(b, d, f)** of unlabelled RGd2 ESCs gated out from mixed cultures with mKO RGd2 (gray) or mKO ETR-KO ESC (light red).
(E) GFP profiles for ETR-KO cells with Doxycline inducible Etv5-p2A-Tcf3 (iEp2aT) transgene expression.
(F) RT-qPCR at 52h post-2i withdrawal and Doxycycline (dox) addition (100ng/ml). Error bar show SD from 2 qPCR replicates.
(G) IF images for NANOG at 3 days post-2i withdrawal and Doxycycline (dox) addition (100ng/ml) taken using a 10x objective.

Figure S6. ETV5 association with transcriptionally dynamic genes (**Related to Fig 7)**
(A) Distribution of unique ETV5 ChIP-seq peaks in 2i and N16h samples. See STAR Methods for peak selection and classification.
(B) Heatmap for relative expression of genes associated with ETV5 that are up- and downregulated in 2i.
(C) Heatmap for ETV5 associated genes upregulated at 16h post-2i withdrawal (N16h). Only genes that show a fold change of ≥1.5 in the UP direction and ≤0.66 in the DOWN direction

in *Etv5*-KO over RGd2 are considered. Expression shown as the log2 difference to the mean across all samples.
(D) Venn diagrams showing the intersection of TCF3 and RBPJ targets with ETV5-bound, upregulated genes in *Etv5*-KO ESCs. Pluripotency regulators are shown in boxes and naïve pluripotency factors are in bold case.

Figure S7. ETV5 association with transcriptionally dynamic genes (Related to Fig7)
(A) RT-qPCR for *Otx2* in parental RGd2 and *Etv5*-KO ESCs upon 2i withdrawal, h=hour. Relative expression normalized to GAPDH is shown. Error bars show SD from 2 wells of the same ESC line cultured in parallel.
(B) ChIP-qPCR for ETV5 binding on putative enhancers shown in Fig 7C-E. ChIP was performed in duplicate (1-2) on ETV5-C-3xFlag knock-in RGd2 cells using an anti-Flag antibody. Error bars indicate SD from 2 qPCR replicates.
(C) Western blot for POU3F1 and LEF1 in RGd2 ESC and *Pou3f1*/*Lef1* single and double knock-out clonal ESC lines. Black line indicates where two halves of the blot were merged after removal of two lanes from a mistargeted clone.
(D) GFP profiles in N2B27 at 26h post-2i withdrawal (N26h).
(E) RT-qPCR at 48h post-2i withdrawal. Error bars show SD from 2 qPCR replicates. Data from 2 independently derived *Lef1/Pou3f1*-dKO (LP-dKO) clonal lines are presented.
(F) UCSC Genome browser tracks showing normalized ChIP-seq read coverage for Etv5 and H3K4me3 (this study), p300, H3K27Ac and H3K4me1 (Buecker *et al* , 2014)
(G) RT-qPCR for Oct4 in 2iLIF and EpiLC on RGd2 and Etv5-KO ESCs (2 independent clonal lines). Error bars show SD from 2 technical replicates for qPCR.
(H) Mean read coverage for p300, H3K27Ac and H3Kme1 (from Buecker et al, 2014) on promoter-associated ETV5-bound loci. Read depth scaled to 1x.

Supplemental Tables

Supplemental Table S1. Differentially expressed genes in *Rbpj* mutants and intersection with genes repressed by RBPJ and TCF3 (Related to Figures 2 and S4)
Supplemental Table S2. Expression of all genes in the RNA-seq datasets (Related to Figure 5)
Supplemental Table S3. Differentially expressed genes in ETR-KO Np5 vs. RGd2 2ip5 (Related to Figure 5)
Supplemental Table S4. Summary of phenotypes in chimeric embryos (Related to figure 6)
Supplemental Table S5. ETV5-associated genomic loci (Related to Figure 7)
Supplemental Table S6. Differentially expressed genes in *Etv5* mutants and intersection with genes regulated by ETV5, RBPJ and TCF3 (Related to Figures 7 and S6)
Supplemental Table S7. Reagents (Related to STAR Methods)

Figure S1 (Related to Figure 1)

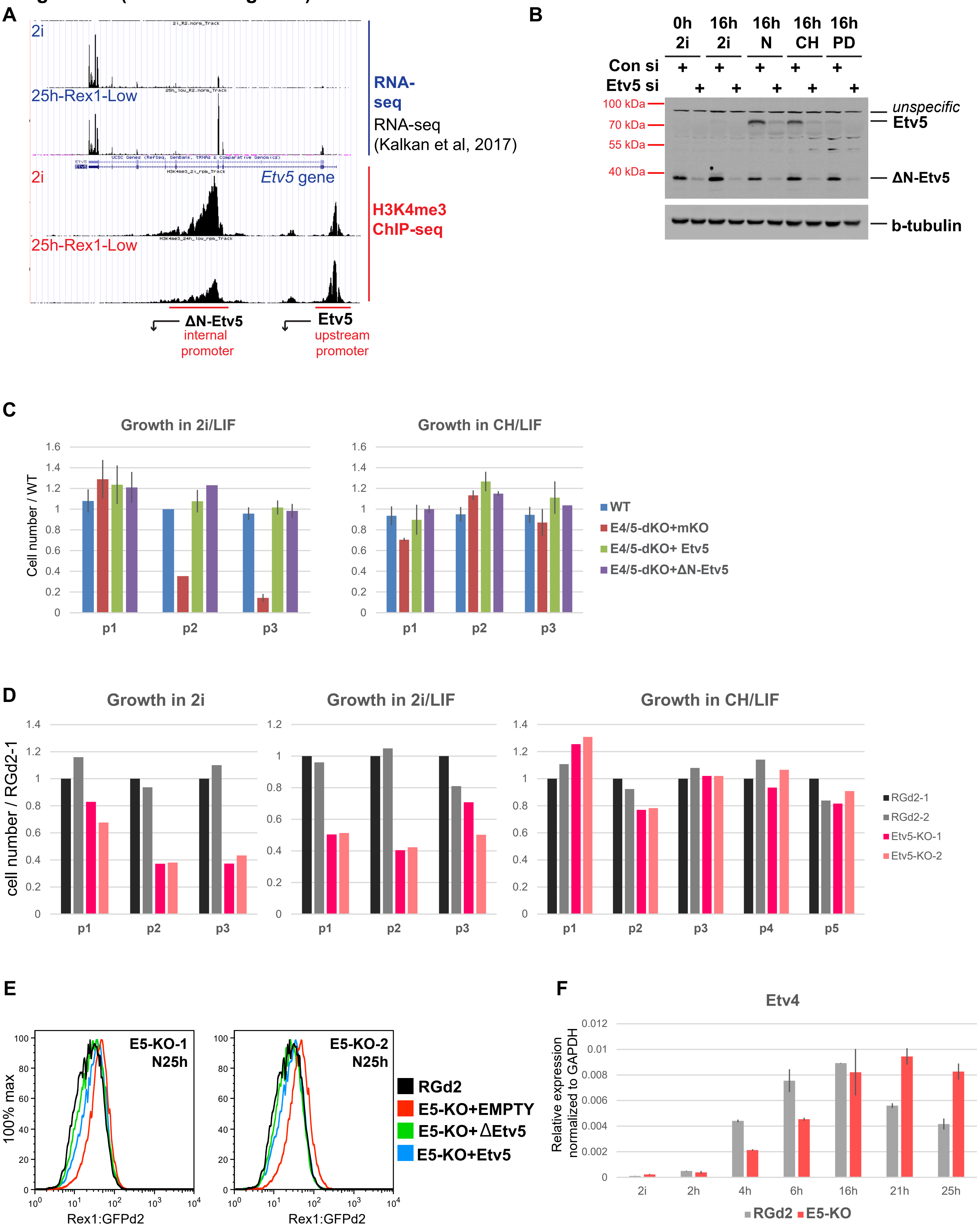

Figure S2 (Related to Figures 1 and 2)

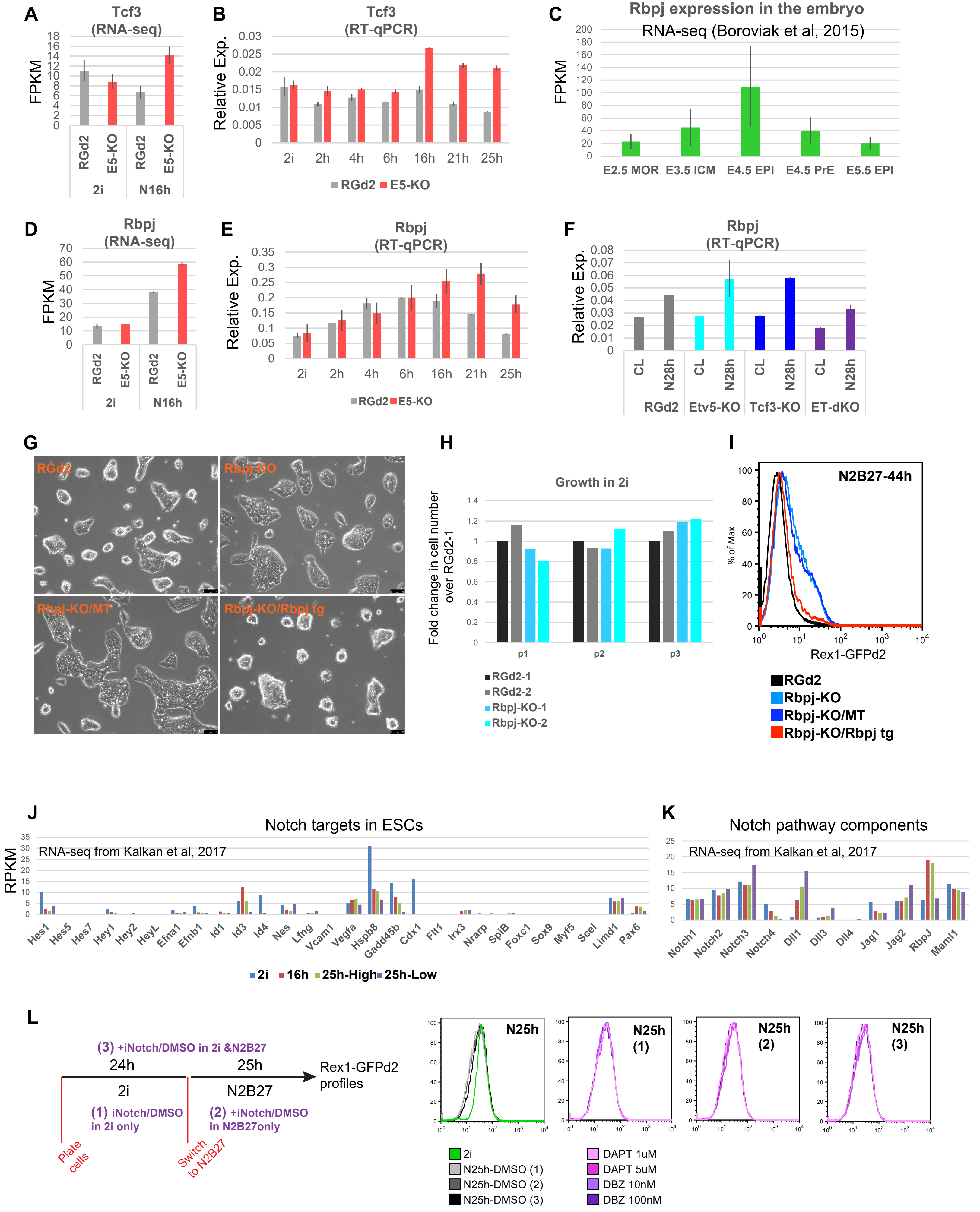

Figure S3 (Related to Figure 2)

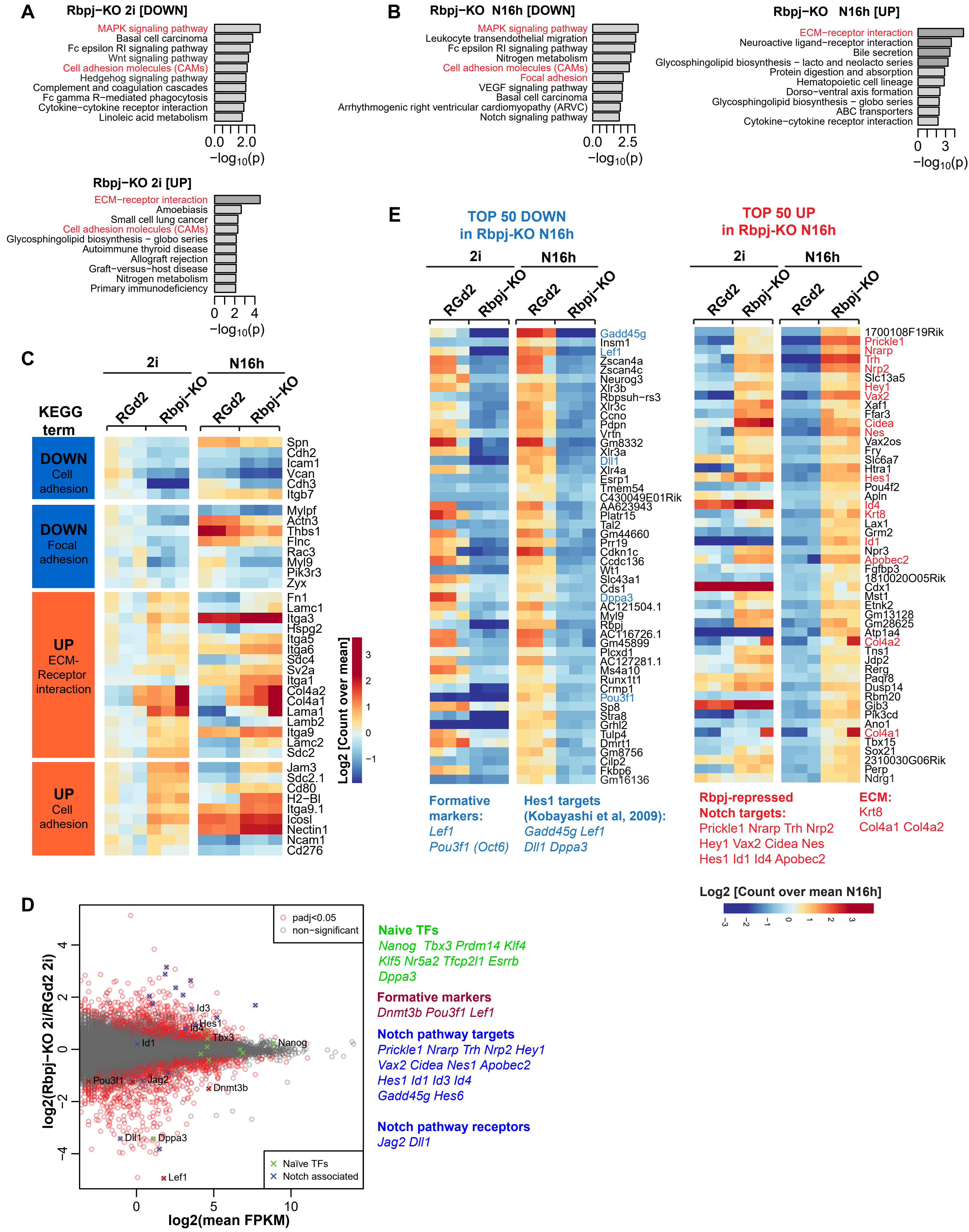

Figure S4 (Related to Figures 2 and 3)

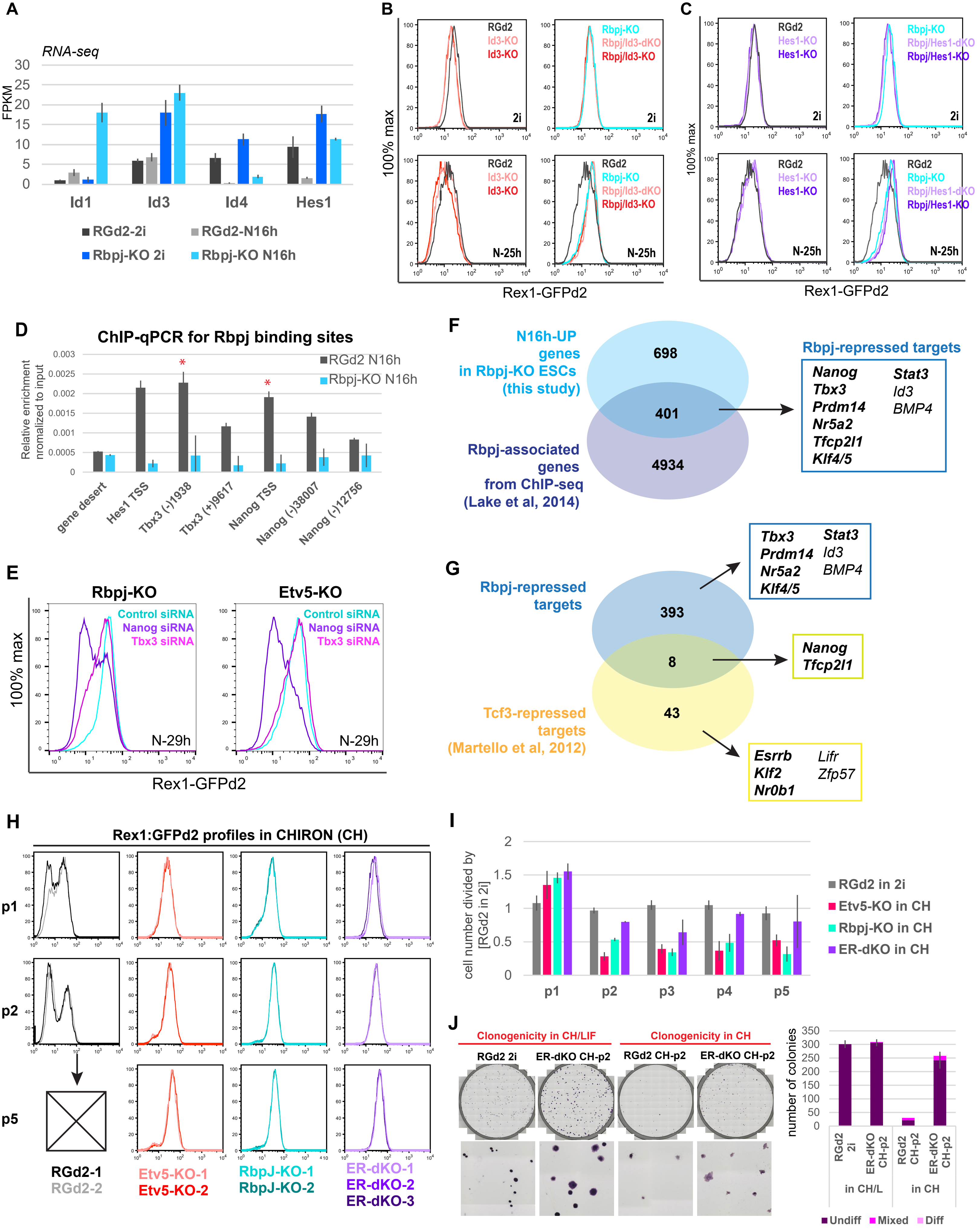

Figure S5 (Related to Figure 3)

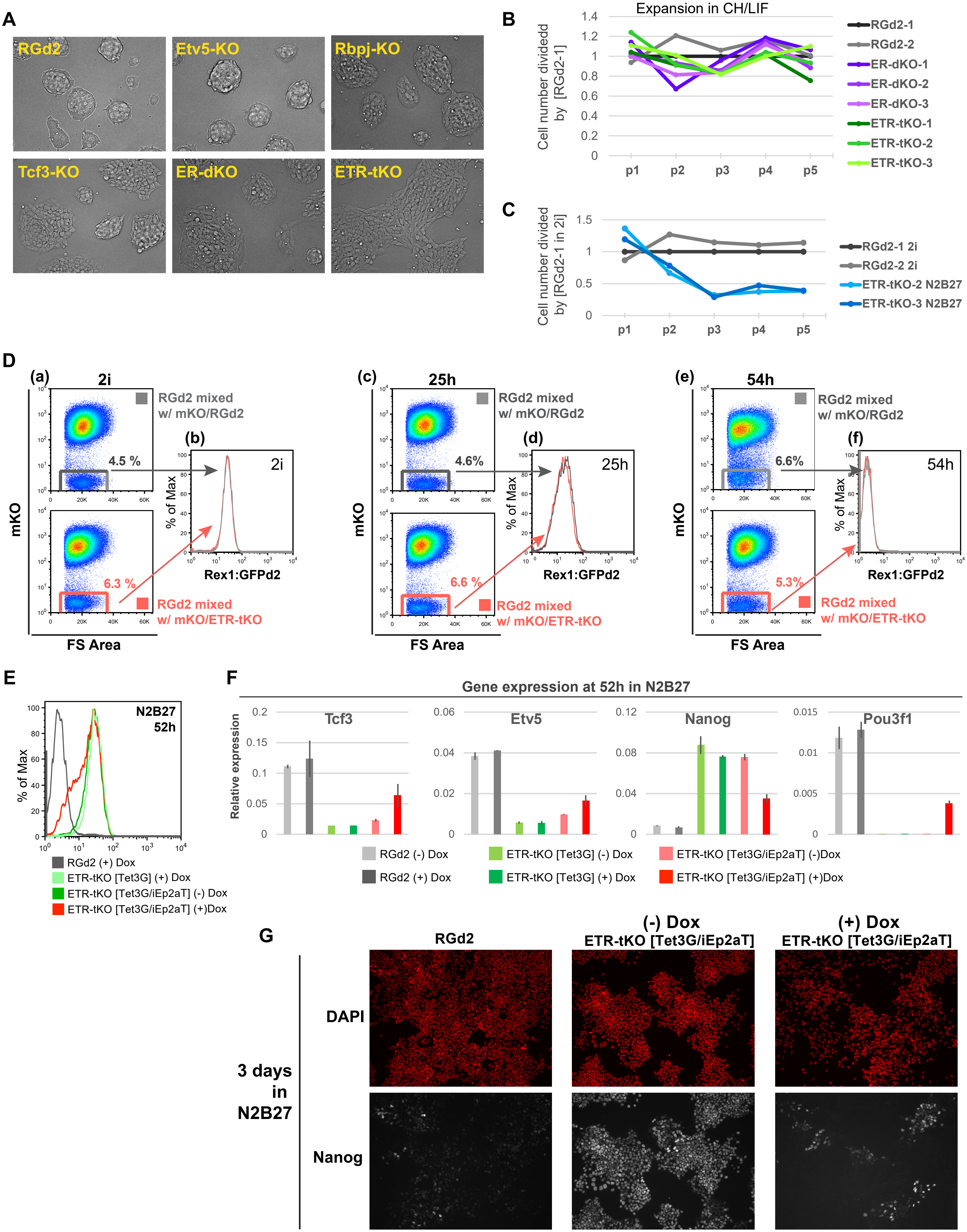

Figure S6 (Related to Figure 7)

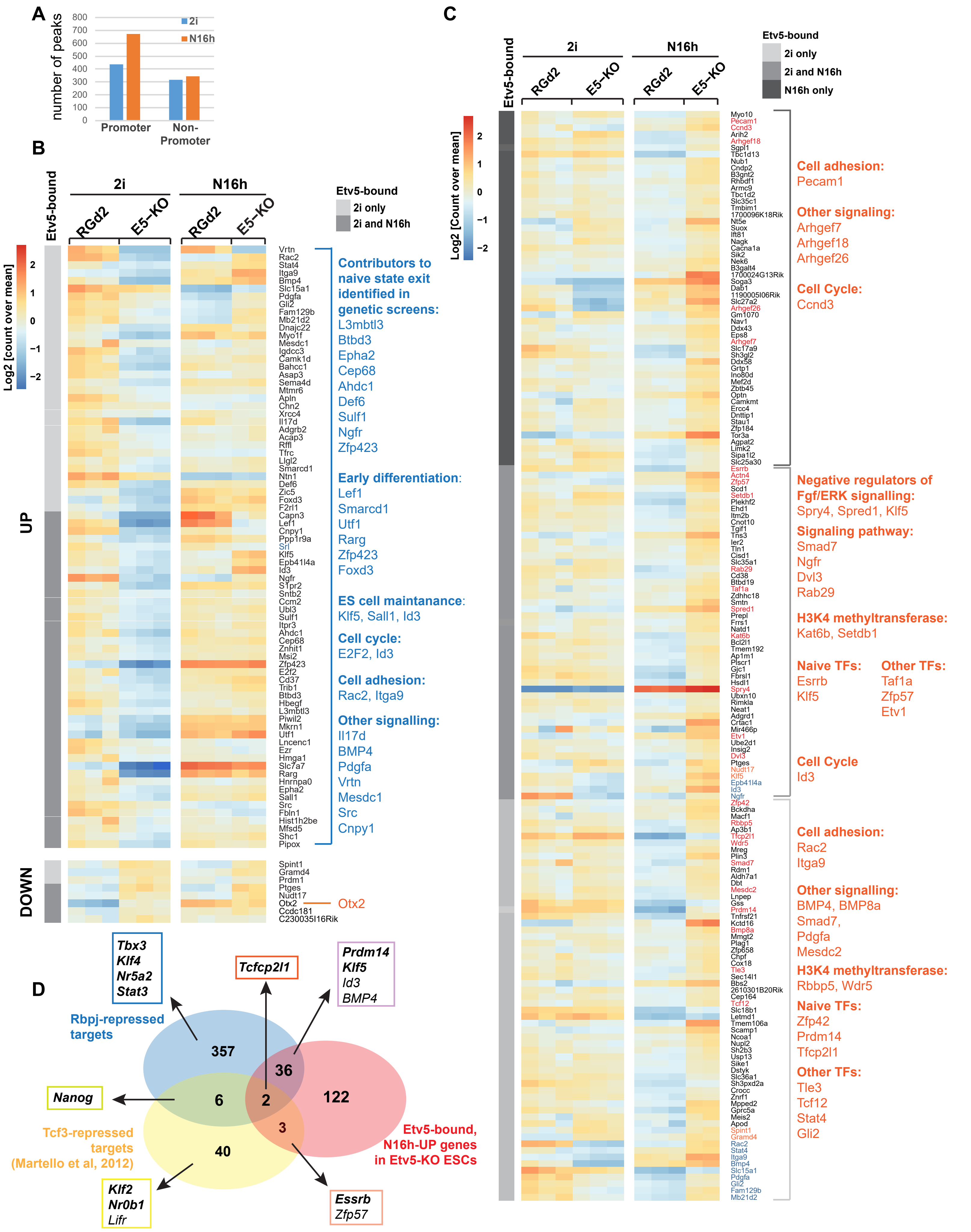

Figure S7 (Related to Fig 7)

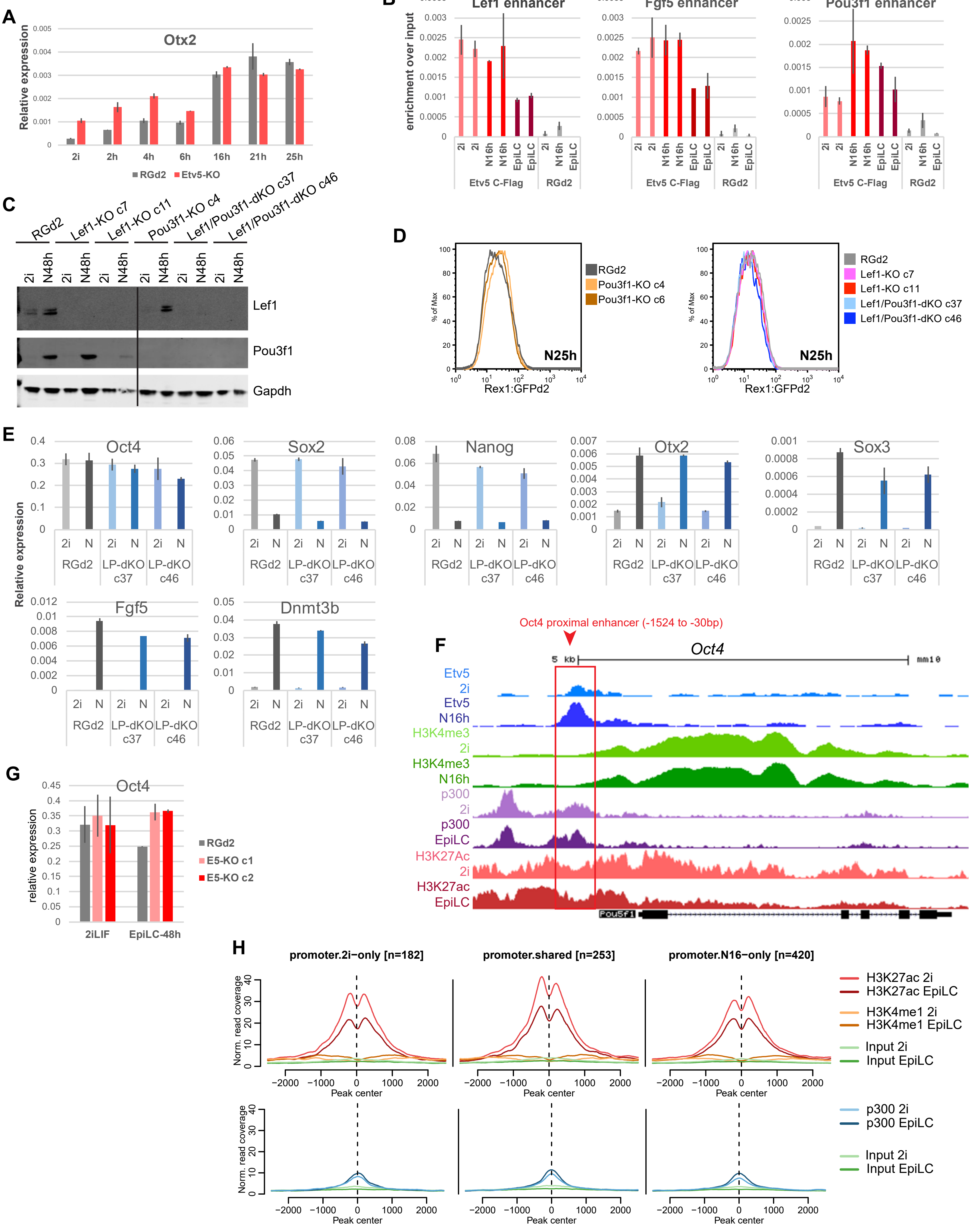

Table S4 Classification of chimaeras

Stage analysed	Injected cell genotype	Embryos injected	Embryos transferred (recipients)	Embryos recovered	Chimeras	Description / Classification
E4.5	WT	5	NA	NA	100% (5/5)	High contribution exclusively to ICM
	KO	9	NA	NA	100% (9/9)	High contribution exclusively to ICM
E6.5	WT	5	5 (1)	100 % (5/5)	100% (5/5)	High contribution to epiblast (5/5);<br>None Rex1 positive;<br>All with posterior T expression;<br>Normal: 5/5<br>Retarded: 0/5<br>Abnormal: 0/5
	KO	10	9 (1)	77.78% (7/9)	100% (7/7)	Moderate contribution to epiblast (7/7);<br>Rex1 signal in majority of injected cells (7/7);<br>T expression (1/7, weak);<br>Contribution extending across embryonic/ extraembryonic boundary (4/7);<br>Normal: 0/7<br>Retarded: 4/7<br>Abnormal: 3/7
E7.5	WT	16	16 (2)	100 % (8/8)<br>100% (8/8)	81.25% (13/16)	High contribution of injected cells (13/13);<br>No Nanog signal detected in 16/16 embryos;<br>Pou3f1 signal in anterior epiblast (8/8);<br>T signal in the primitive stream region (8/8);<br>Normal: 16/16<br>Retarded: 0/16<br>Abnormal: 0/16
	KO	44	44 (4)	0% (0/11)<br>0% (0/11)<br>54.55% (6/11)<br>63.64% (7/11)	100% (13/13)	Recipient failure for 2/4 hosts;<br>Mild to moderate contribution of injected cells (13/13);<br>Detached Reichert's membrane (10/13);<br>High Nanog in majority of injected cells (13/13);<br>scattered Pou6f1 signal in the epiblast (7/7), but not in injected cells;<br>T expression (0/6);<br>Normal: 0/13<br>Retarded: 0/13<br>Abnormal: 13/13

Normal: No apparent developmental defects
Abnormal: Malformed embryos
Retarded: Small, but normal morphology

All chimaeras were generated by microinjection of 8 cells into E2.5 embryos.
